# Regulation of mitochondrial metabolism by autophagy supports leptin-induced cell migration

**DOI:** 10.1038/s41598-024-51406-y

**Published:** 2024-01-16

**Authors:** Alin García-Miranda, José Benito Montes-Alvarado, Fabiola Lilí Sarmiento-Salinas, Verónica Vallejo-Ruiz, Eduardo Castañeda-Saucedo, Napoleón Navarro-Tito, Paola Maycotte

**Affiliations:** 1 Laboratorio de Biología Celular del Cáncer, Facultad de Ciencias Químico Biológicas, Universidad Autónoma de Guerrero, 39090 Chilpancingo de los Bravo, Guerrero Mexico; 2 Laboratorio de Bioquímica Metabólica, Centro de Investigación Biomédica de Oriente, Instituto Mexicano del Seguro Social, 74360 Atlixco, Puebla Mexico; 3 Consejo Nacional de Humanidades, Ciencias y Tecnologías, 03940 Ciudad de México, Mexico; 4 Laboratorio de Biología Molecular, Centro de Investigación Biomédica de Oriente, Instituto Mexicano del Seguro Social, 74360 Atlixco, Puebla México

**Keywords:** Cancer, Breast cancer, Autophagy, Cell growth, Cell migration, Cell signalling

## Abstract

Leptin is an adipokine secreted by adipose tissue, which promotes tumor progression by activating canonical signaling pathways such as MAPK/ERK. Recent studies have shown that leptin induces autophagy, and this process is involved in leptin-induced characteristics of malignancy. Autophagy is an intracellular degradation process associated with different hallmarks of cancer, such as cell survival, migration, and metabolic reprogramming. However, its relationship with metabolic reprogramming has not been clearly described. The purpose of this study was to determine the role of leptin-induced autophagy in cancer cell metabolism and its association with cellular proliferation and migration in breast cancer cells. We used ER^+^/PR^+^ and triple-negative breast cancer cell lines treated with leptin, autophagy inhibition, or mitochondrial metabolism inhibitors. Our results show that leptin induces autophagy, increases proliferation, mitochondrial ATP production and mitochondrial function in ER^+^/PR^+^ cells. Importantly, autophagy was required to maintain metabolic changes and cell proliferation driven by leptin. In triple-negative cells, leptin did not induce autophagy or cell proliferation but increased glycolytic and mitochondrial ATP production, mitochondrial function, and cell migration. In triple negative cells, autophagy was required to support metabolic changes and cell migration, and autophagy inhibition decreased cellular migration similar to mitochondrial inhibitors. In conclusion, leptin-induced autophagy supports mitochondrial metabolism in breast cancer cells as well as glycolysis in triple negative cells. Importantly, leptin-induced mitochondrial metabolism promoted cancer cell migration.

## Introduction

According to the latest statistics worldwide, more than 100,000 breast cancer cases in postmenopausal women were attributed to excess body mass^1^. Biologically, adipose tissue has endocrine functions that regulate physiological processes associated with appetite regulation and energy balance^2^. However, during body weight gain, the adipose tissue experiments several changes in function with alterations in the secretion of multiple biomolecules considered as pro-tumorigenic, such as leptin^3^. Elevated plasma leptin levels (> 20 ng/mL) have been associated with increased risk, aggressiveness, and recurrence of breast cancer^4^, and several mechanisms by which leptin induces tumor progression have been described. Some of them involve activating signaling pathways that allow pro-tumoral gene expression^5^, while others include regulating intracellular processes, such as autophagy and energy metabolism^6^.

Autophagy is an intracellular process active at low levels on physiological conditions, responsible for the degradation of bulk cytoplasmic components and nutrient recycling, thus maintaining cellular homeostasis and survival^7^. Under stress conditions, autophagy is induced and removes damaged organelles, proteins, and lipids, which in autophagy deficient conditions would accumulate and promote genomic instability eventually progressing into cancer^8^. Therefore, in a healthy organism, autophagy is considered a tumor suppressor process, in contrast to occasions where a tumor has formed, and where autophagy favors tumorigenic characteristics^9^. Since autophagy in cancer has been mostly described as a pro-tumoral process, strategies for its manipulation have been implemented with a therapeutic approach for several types of cancer, including breast cancer^10^. However, the outcome of autophagy modulation in clinical trials has not been conclusive.

Adenosine 5′-triphosphate (ATP) is the main energy resource in animal cells, resulting from the breakdown of glucose via glycolysis in the cytoplasm or by oxidative metabolism in mitochondria^11^. In terms of efficiency, mitochondrial oxidative metabolism produces an ATP yield at least 14.5 higher than glycolysis, and both processes are usually coupled and contribute to energy production in healthy cells^11^. Conversely, tumor cells frequently exhibit a dominance of glycolysis over mitochondrial metabolism, a phenomenon called the Warburg effect and recognized as metabolic reprogramming^12^. Although the Warburg metabolic profile predominates in different tumors, the presence of functional mitochondria capable of producing ATP has been evidenced in cancer cells^13^. Additionally, throughout tumor progression, changes in the microenvironment and the cancer cell metabolic needs, suggest a reverse shift in the metabolic profile with a predominance of mitochondrial oxidative metabolism in advanced stages of cancer^14^. This metabolic shift has become an attractive target for identifying new therapeutic approaches regulating metabolism. Although different inhibitors targeting mitochondrial metabolism and glycolytic enzymes have been proposed, studies in different tumor contexts are lacking^15–17^.

Given the diversity of substrates that can be degraded by autophagy, this process is closely linked to cell metabolism and is critical to the maintenance of the cellular energetic state, especially during stressful conditions occurring during metastasis^18^. For this reason, autophagy might support the metabolic compensation occurring during cancer progression, and also the combination of autophagy and metabolic inhibitors could prevent adaptive responses of cancer cells or the tumor microenvironment^18^. Previous reports in breast cancer cells suggest that autophagy is required to sustain leptin-driven Extracellular-Signal-Regulated-Kinase phosphorylation (ERK), cellular proliferation and migration^19–21^. Although the specific molecular mechanisms are unknown, the evidence suggests that autophagy is required to maintain an optimal mitochondrial metabolism^22^, which could eventually sustain the metabolic demand of tumor processes such as cellular proliferation or migration. Therefore, the aim of this study was to evidence the role of autophagy in cancer cell metabolism and to define its relationship with the proliferation and migration of breast cancer cells exposed to leptin.

## Results

### Leptin treatment affected cellular morphology and increased ERK activation in breast cancer cells

**Figure 1:**
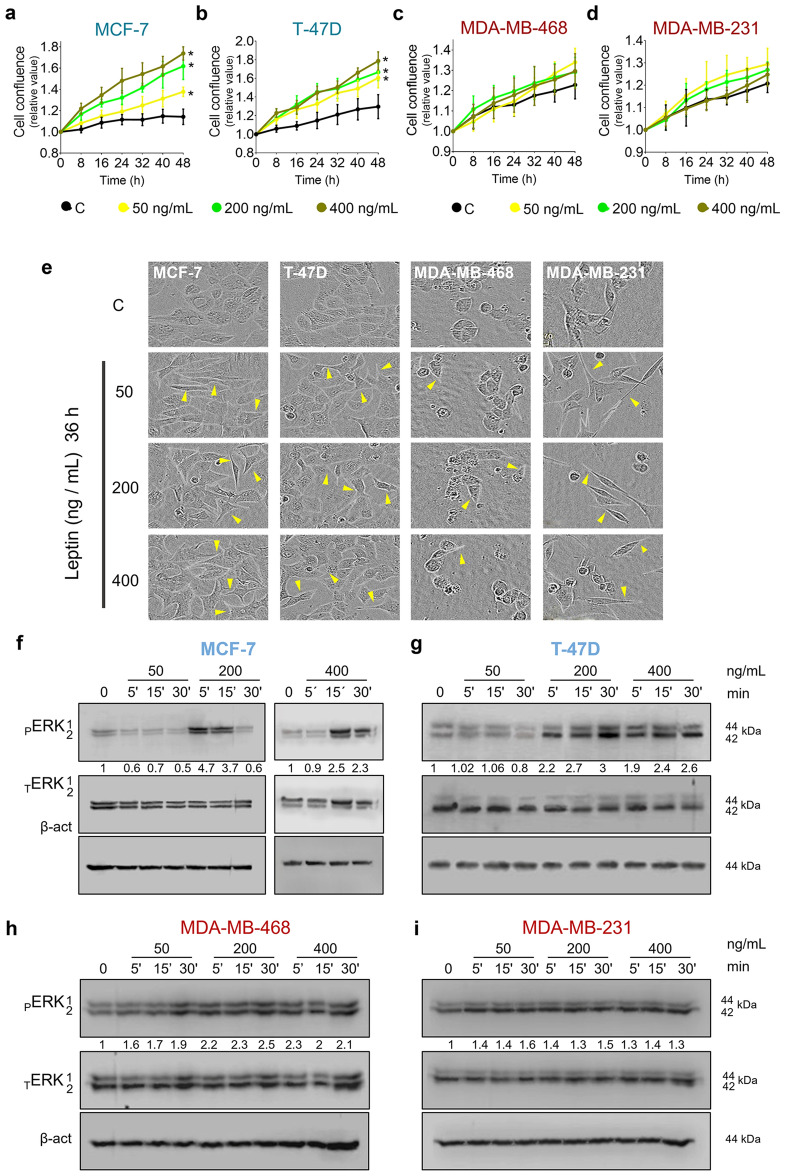
Effect of different leptin concentrations on proliferation, morphology and ERK phosphorylation in breast cancer cells. Cells were treated with different leptin concentrations and monitored by microscopy every 8 h to determine changes in cell confluence during 48 h (**a–d**). Leptin increased cell proliferation in ER^+^/PR^+^ (**a–b**) cells but not in triple-negative cells (**c–d**). Leptin-induced changes in cell morphology are indicated by yellow arrows (**e**). Western blots reflect the changes in leptin-mediated ERK phosphorylation (**f–i**). ERK phosphorylation increased in ER^+^/PR^+^ cells at 200 and 400 ng/mL of leptin (**f–g**). On the other hand, on triple-negative cells all leptin concentrations increased ERK phosphorylation similarly (**h–i**). The numbers below the blot represent the normalized quantification of ERK1 and ERK2 phosphorylation, with respect to total ERK and β-actin. The original blots are shown in Supplementary Figure S12. The control was treated with a vehicle. C: control. Graphs show mean ± S.D.; n = 2 in quadruplicate. One-way ANOVA; Tukey post hoc. *p* < 0.05. * vs. C.

It is well recognized that leptin acts as a pro-tumoral agent. However, breast cancer cells respond differently depending on leptin concentration^23–25^. Therefore, we assessed the effect of three concentrations of leptin (50, 200, and 400 ng/mL) on cell proliferation, cell morphology, and ERK phosphorylation of breast cancer cell lines. As shown in Fig. 1, leptin had a differential effect on breast cancer cell proliferation. In hormone receptor-positive MCF-7 and T-47D (ER^+^/PR^+^) cells, all leptin concentrations significantly increased proliferation (Fig. 1a,b), while in triple-negative MDA-MB-468 and MDA-MB-231 cells, leptin did not affect cellular proliferation (Fig. 1c,d) or death (Supplementary Fig. S1). Interestingly, all leptin concentrations induced changes in cell morphology (Fig. 1e). Under control conditions, ER^+^/PR^+^ cells exhibited colony growth pattern, cell–cell junctions, and epithelial morphology (Fig. 1e). MDA-MB-468 cells have epithelial morphology, cell–cell junctions, and grow with a grape cluster-like pattern, and MDA-MB-231 cells have mesenchymal morphology and individual growth pattern (Fig. 1e). After leptin treatment, ER^+^/PR^+^ cells acquired a fibroblastoid-like shape which was more evident as leptin concentration increased (Fig. 1e). On the other hand, MDA-MB-468 cells lost cell–cell junctions and generated conical-shaped membrane extensions (Fig. 1e). In contrast, MDA-MB-231 cells generated long needle-shaped membrane extensions (Fig. 1e). In both triple-negative breast cancer cell lines, the change in morphology was similar with all leptin concentrations used. Regarding cellular signaling induced by leptin, most leptin concentrations increased ERK phosphorylation in all the cell lines tested (Fig. 1f - i). In ER^+^/PR^+^ cells, ERK phosphorylation increased with 200 and 400 ng/mL of leptin (Fig. 1f, g), whereas, in triple-negative cells, the treatment with 50 ng/mL of leptin was enough to increase ERK phosphorylation (Fig. 1h, i). Taken together, our data suggest that leptin differentially influences tumor characteristics of breast cancer cells. In ER^+^/PR^+^ cells, the effect seems to be dose-dependent since gradual changes in proliferation, cell morphology, and ERK phosphorylation were observed as leptin concentrations increased (Fig. 1a,b,e–g). Conversely, in triple-negative cells, exposure to the lowest concentration of leptin was sufficient to promote changes in cell morphology (Fig. 1c–e) and ERK phosphorylation, which did not increase at higher leptin concentrations (Fig. 1h,i). Other pathways activated by leptin include JAK2/STAT3 and PI3K/AKT signaling^5^. We found increased phosphorylation of JAK2 and AKT starting at the 50 ng/mL dose in the triple-negative cell lines (Supplementary Fig. S2), indicating that this concentration was enough to induce leptin canonical signaling pathways, thus we selected 50 ng/mL for further experiments in triple-negative cells. In ER^+^/PR^+^ cells, leptin induced JAK2 and AKT phosphorylation in the T-47D but not in the MCF-7 cell line (Supplementary Fig. S2). Since leptin induced AKT and ERK phosphorylation starting at 200 ng/mL (Supplementary Fig. S2), and all the concentrations tested induced proliferation (Fig. 1a,b), we selected the highest leptin concentration for ER^+^/PR^+^ cells for the next experiments.

### Leptin had a differential effect on the promotion of autophagy in breast cancer cells

**Figure 2:**
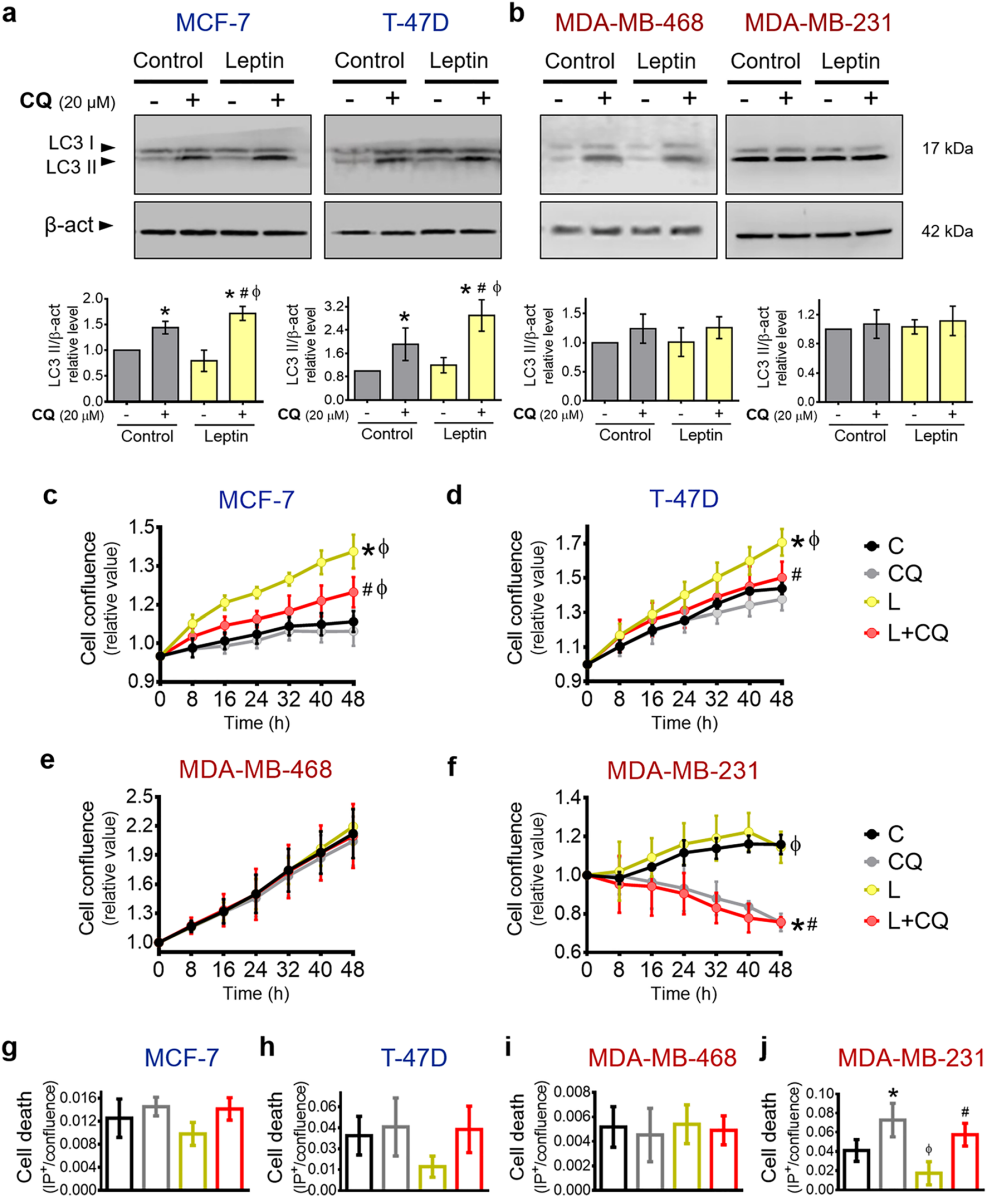
Effect of autophagy on the proliferation of breast cancer cells treated with leptin. Leptin treatment induced autophagy in ER^+^/PR^+^ (**a**) but not in triple-negative cells (**b**). Leptin-induced autophagy in ER^+^/PR^+^ supported the increase in proliferation induced by leptin (**c–d**) without changes in cell death (**g–h**). The proliferation or cell death of MDA-MB-468 triple-negative cells was not affected by leptin or autophagy inhibition (**e,i**), and autophagy inhibition reduced cell proliferation (**f**) and increased cell death (**j**), independent of leptin treatment in MDA-MB-231 triple-negative cells. ER^+^/PR^+^ cells were treated with 400 ng/mL leptin (**a,c,d,g,h**) and triple-negative cells with 50 ng/mL leptin (**b,e,f,i,j**) as specified in the “Methods” section. For all assays, chloroquine (CQ) was used at 20 μM. To evaluate changes in autophagy by western blotting, CQ was added two hours before the end of the 24 h treatment with or without leptin (**a,b**). For proliferation and cell death assays leptin and CQ were added during 48 h (**c–j**). The original and complete blots are shown in Supplementary Figures S13 and S14. The control was treated with leptin vehicle. C: control; L: leptin; CQ: chloroquine. Graphs show mean ± S.D; WB: n = 4; proliferation and cell death: n = 4 in quadruplicate; Two-way ANOVA; Tukey post hoc; *p* < 0.05. * vs. C; # vs. L; ɸ vs CQ.

It has been reported that leptin can modify the process of autophagy and favor leptin-driven tumor development and growth^26^. To demonstrate the involvement of autophagy in some of the leptin-induced cancer characteristics, we first determined the effect of leptin on autophagy in breast cancer cells. Changes in autophagy were assessed by changes in the amount of LC3 II (incorporated into autophagosome membranes) in the absence or presence of the autophagy inhibitor chloroquine (CQ), which prevents autophagosome degradation and allows their accumulation, reflected by an increase in LC3 II^27^. As shown in Fig. 2a, in ER^+^/PR^+^ cells, leptin did not change LC3 II levels when compared to control cells. However, when CQ was added to leptin-treated cells, LC3 II significantly increased when compared to CQ treated cells, indicating that leptin induced autophagy in hormone receptor-positive breast cancer cells.

On the other hand, in triple-negative breast cancer cells, there was no difference in LC3 II between leptin/CQ treatment compared to the CQ-treated control (Fig. 2b), indicating that leptin did not induce autophagy in triple-negative cells. These results demonstrate that leptin shows a distinct effect on autophagy in breast cancer cells. Leptin induced autophagy in hormone receptor-positive cells but not in triple-negative breast cancer cells.

Autophagy is a process highly associated with tumor cell survival and proliferation. Since leptin had a differential effect on autophagy (Fig. 2a,b), we evaluated the role of autophagy in cell proliferation under the effect of leptin treatment during 48 h. As expected, leptin increased the proliferation of ER^+^/PR^+^ cells (Fig. 2c,d) but not in triple-negative cells (Fig. 2e,f) when measured by real time-microscopy or by CFSE staining and flow cytometry (Supplementary Fig. S3). Interestingly, inhibition of autophagy with CQ treatment in cells treated with leptin significantly reduced leptin-induced proliferation in ER^+^/PR^+^ cells (Fig. 2c,d) without changes in cell death (Fig. 2g,h, Supplementary Fig. S4). Interestingly, although leptin did not induce autophagy in triple-negative cells, both cell lines had different responses to autophagy inhibition. In MDA-MB-468 cells, autophagy inhibition did not modify proliferation (Fig. 2e, Supplementary Fig. S3) or cell death (Supplementary Fig. S4), in the presence of leptin (Fig. 2i). Conversely, in MDA-MB-231 cells, autophagy inhibition significantly reduced cell proliferation (Fig. 2f, Supplementary Fig. S3) and increased cell death (Fig. 2j, Supplementary Fig. S4) independently of leptin treatment. These results suggest that triple-negative breast cancer cell proliferation is not leptin-dependent and that in highly invasive mesenchymal cells with a claudin-low profile, such as MDA-MB-231 cells, survival depends on basal autophagy in control or leptin-treated conditions.

Taken together, these data support that in ER^+/^PR^+^ cells, leptin-induced autophagy was required to sustain leptin-driven proliferation, while in triple-negative cells, leptin did not induce or change the levels of basal autophagy neither affected proliferation. Nevertheless, MDA-MB-231 cells were highly dependent on the autophagy process to survive in the presence or absence of leptin.

### Autophagy sustains leptin-induced increase in ATP production in breast cancer cells

It has been recognized that cancer cells preferentially exhibit a glycolytic metabolic profile to drive macromolecule synthesis and cell growth^28^. However, tumor cells can adapt their metabolic demands for long periods of time as a response and adaptation to tissue micro-environmental conditions^14^. In metabolic remodeling, autophagy has been proposed as a critical process that impacts multiple metabolic pathways and allows resolution of energetic stress throughout tumor progression^18^. Accordingly, we hypothesized that autophagy could regulate the energetic metabolic profile of leptin-treated breast cancer cells to sustain tumor processes such as cellular proliferation or migration. Thus, we assessed the rate of ATP production by distinguishing between fractions produced by glycolysis or the mitochondria. We measured changes in oxygen consumption (OCR) and extracellular acidification (ECAR) during a serial injection of metabolic modulators (oligomycin, an ATP synthase inhibitor; rotenone/antimycin A, mitochondrial complex I and III inhibitors) which allows calculating the ATP production rate.

**Figure 3:**
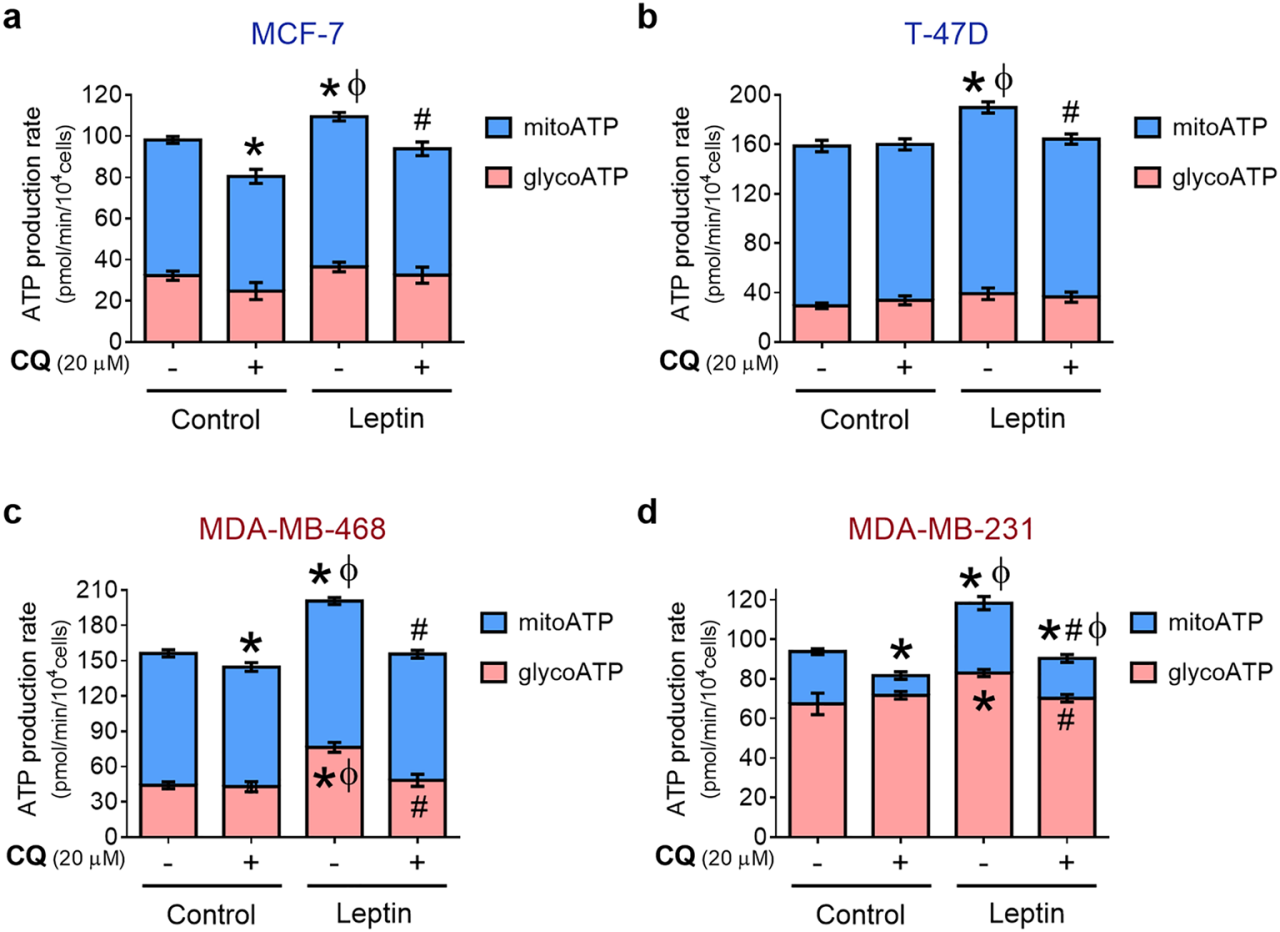
Effect of autophagy on energy metabolism of leptin-treated breast cancer cells. Leptin increased mitochondrial ATP in ER^+^/PR^+^ cells (**a–b**) and triple-negative cells (**c–d**); leptin treatment also increased glycolytic ATP in triple-negative cells (**c–d**). Autophagy inhibition prevented the increase in mitochondrial ATP induced by leptin in all breast cancer cells and the increase in glycolytic ATP production in triple negative breast cancer cells. ER^+^/PR^+^ cells were treated with 400 ng/mL leptin (**a–b**) and triple-negative cells with 50 ng/ml leptin (**c–d**) for 24 h. CQ was used at 20 μM in all cell lines for 24 h. Oligomycin and rotenone/ antimycin A were used to assess mitochondrial and glycolytic ATP. The control in all experiments was treated with a vehicle. CQ: chloroquine. Graphs show mean ± S.D.; n = 3 in triplicate; two-way ANOVA; Sidak post hoc; p < 0.05. * vs C; # vs L; ɸ vs CQ.

Cells were treated with leptin and/or CQ for 24 h and then assessed for ATP production rate in a Seahorse XFe 96 analyzer. Figure 3 shows that breast cancer cells have a different basal energetic profile. In control conditions, ER^+^/PR^+^ cells (Fig. 3a,b) and MDA-MB-468 triple-negative cells (Fig. 3c) had a higher mitochondrial than glycolytic ATP production. Oppositely, the major ATP source for MDA-MB-231 triple-negative cells was glycolysis (Fig. 3d). When cells were treated with leptin, we observed an increase in total ATP and mitochondrial ATP production in all cell lines (Fig. 3a–d). Interestingly, leptin treatment also increased glycolytic ATP, but only in triple-negative cells (Fig. 3c,d). Inhibition of autophagy with CQ in leptin-treated cells significantly reduced mitochondrial ATP in ER^+/^PR^+^ cells (Fig. 3a,b) or mitochondrial and glycolytic ATP production in triple negative cells (Fig. 1c, d). A similar behavior was observed at 48 h (Supplementary Fig. S5). This data indicates that autophagy was required for leptin-driven mitochondrial ATP production in all breast cancer cell lines tested. Autophagy also contributed to leptin-induced glycolytic ATP production but only in triple-negative breast cancer cells.

### Autophagy is required to maintain the leptin-induced enhancement of the mitochondrial function of breast cancer cells

**Figure 4:**
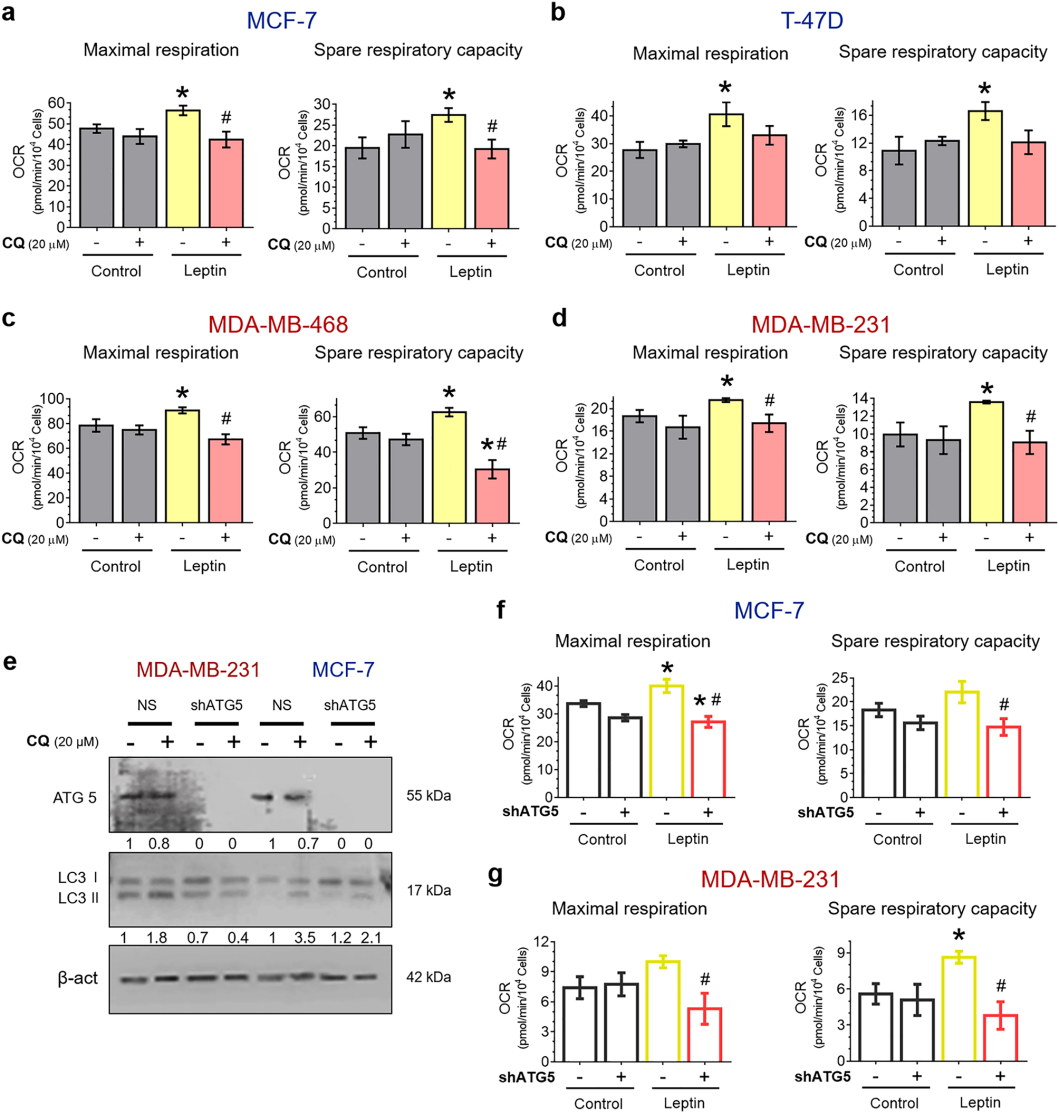
Effect of autophagy on mitochondrial function of leptin-treated breast cancer cells. Leptin increased the maximal respiration and spare respiratory capacity in all breast cancer cells, and autophagy inhibition with CQ reduced leptin-induced changes in mitochondrial function (**a–d**). The blot shows an effective knockdown of ATG5 of cells grown in full media in both conditions, resulting in effective inhibition of autophagy as measured by LC3 II accumulation in MCF-7 and MDA-MB-231 cells (**e**). In non-silencing cells (NS), leptin increased the maximal respiration and spare respiratory capacity (**f–g**) and ATG5 knockdown (shATG5) prevented the enhancement in mitochondrial functions induced by leptin (**f–g**). Non-transduced and transduced ER^+^/PR^+^ and triple-negative cells were treated with 400 ng/mL and 50 ng/mL of leptin respectively for 24 h (**a–g**). For autophagy inhibition, CQ was used at 20 µM for 24 h in metabolic assay (**a–d**;**f,g**), but also for the last 2 h to measure autophagy flux after ATG5 knockdown (**e**). Then, metabolic measurements were performed with a Mito Stress kit: oligomycin, FCCP, and rotenone/antimycin A. The numbers below the blot represent the normalized quantification of ATG5 and LC3 II respect to β-actin. The original and complete blots are shown in Supplementary Figure S15. The control was treated with a vehicle. CQ: chloroquine; NS: non silencing shRNA; shATG5: shRNA silencing ATG5. mean ± S.D.; a-d: n = 3 in quadruplicate. f-g: n = 2 in triplicate; one-way ANOVA. Tukey and Dunnett post hoc; *p* < 0.05. * vs C; # vs L.

Since autophagy was required for mitochondrial ATP production in breast cancer cells, we evaluated the role of autophagy on parameters associated with mitochondrial function, such as ATP-linked respiration, proton leak, basal respiration, maximal respiration, and spare respiratory capacity. This was done by the sequential injection of cellular respiration modulators (oligomycin, rotenone/antimycin A and FCCP, an uncoupling agent that collapses the proton gradient and disrupts the mitochondrial membrane potential), and OCR measurement in a Seahorse XFe 96 metabolic analyzer. Before analyzing mitochondrial function, breast cancer cells were treated with leptin and/or CQ for 24 h. We observed that leptin treatment significantly increased maximal respiration and mitochondrial spare respiratory capacity in all breast cancer cell lines (Fig. 4a–d, yellow bar). Maximal respiration is a parameter that represents the maximum capacity of the electron transport chain (ETC), while spare respiratory capacity reflects the cellular fitness to respond to changes in energetic demands^29^. Interestingly, autophagy inhibition with CQ prevented the increase in maximal respiration and spare respiratory capacity induced by leptin in all breast cancer cells (Fig. 4a–d, red bar), indicating the involvement of autophagy in leptin-enhanced mitochondrial function. Other mitochondrial parameters were found to be affected by leptin treatment such as non-mitochondrial oxygen consumption, or regulated by autophagy, such as basal mitochondrial respiration or mitochondrial ATP production. However, these changes did not reach statistical significance in all the breast cancer cell lines studied (Supplementary Fig. S6).

Next, to support these findings and discard unspecific effects of the pharmacological autophagy inhibitor, CQ, we performed a knockdown of the autophagy-related protein ATG5 in two breast cancer cell lines: MCF-7 hormone receptor-positive cells and MDA-MB-231 triple-negative cells. An effective knockdown of ATG5 was achieved in both cell lines as well as an effective inhibition of autophagy as measured by LC3 II accumulation (Fig. 4e). In control (NS) cells, LC3 II was increased in the presence of CQ, demonstrating a block in basal autophagy. Oppositely, in cells with ATG5 knockdown (shATG5) LC3 II did not accumulate in response to CQ treatment, suggesting a decreased autophagy flux (Fig. 4e). To assess mitochondrial function, first NS and shATG5 cells were treated with leptin for 24 h, and then a metabolic assay was performed. The results show that leptin increased the maximal respiration and spare respiratory capacity in MCF-7 and MDA-MB-231 non-silencing cells (Fig. 4f,g). Interestingly, ATG5 knockdown reduced the enhancement in mitochondrial functions induced by leptin (Fig. 4f,g), indicating that genetic inhibition of autophagy prevented leptin-induced mitochondrial fitness. Similar to CQ treatment, other mitochondrial parameters were affected by leptin treatment such as mitochondrial ATP production or non-mitochondrial oxygen consumption, and others were also affected by the inhibition of autophagy, such as basal mitochondrial respiration or mitochondrial ATP production. However, these parameters did not reach statistical significance in both cell lines evaluated (Supplementary Fig. S7). Energetic parameters affected by leptin treatment which were completely independent of autophagy include non-mitochondrial oxygen consumption (Supplementary fig. S6 and S7), indicating non-mitochondrial oxidation effects induced by leptin. Taken together, these results indicate that autophagy sustains leptin-induced mitochondrial fitness in breast cancer cells despite their different basal metabolic profiles.

### Inhibition of OXPHOS and autophagy avoid leptin-induced migration in invasive breast cancer cells

**Figure 5:**
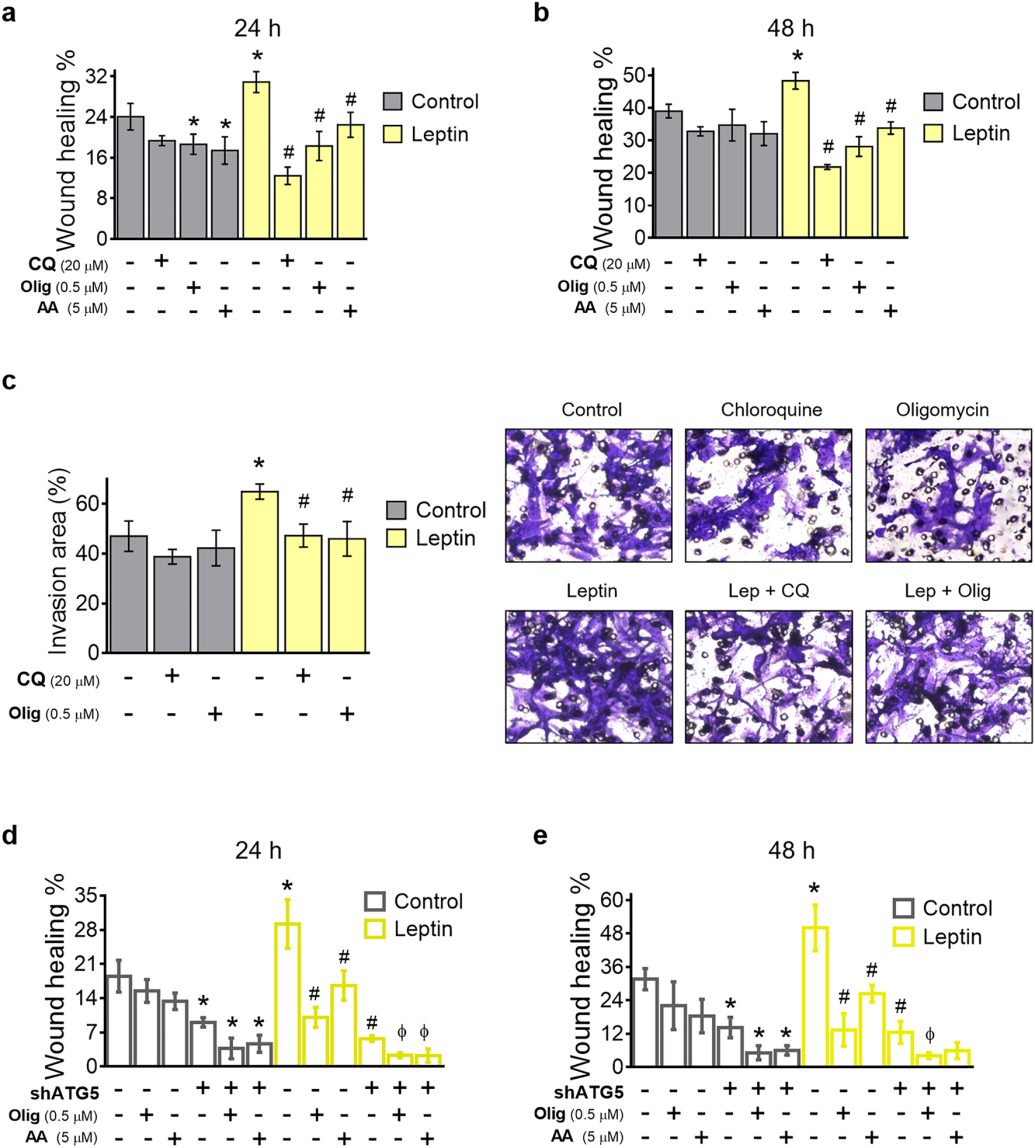
Effect of autophagy inhibition and mitochondrial function on leptin-induced migration in MDA-MB-231 cells. OXPHOS inhibition in MDA-MB-231 cells reduced cell migration at 24 h (**a**). In the presence of leptin, cell migration increased significantly at 24 (**a**) and 48 h (**b**). Also, leptin increased invasion at 48 h (**c**). Leptin-induced cell migration and invasion were prevented by autophagy inhibition with CQ (**a–c**). Similarly, in non-silencing (control) MDA-MB-231 cells, leptin induced cellular migration which was prevented by knockdown of ATG5 (**d,e**). Importantly, basal and leptin-induced cell migration was decreased with the use of mitochondrial inhibitors oligomycin and antimycin A in autophagy knockdown cells (**d,e**). For invasion and migration assay, all cells were treated with leptin 50 ng/mL, CQ, AA, and Olig during 48 h. The control was treated with a vehicle. C: control; L: leptin; CQ: chloroquine; Olig: oligomycin; AA: antimycin A; NS: non silencing; shATG5: ATG5 knockdown. Graphs show mean ± S.D.; (**a,b)**: n = 3 in quadruplicate; (**c)**: n = 2 in triplicate; (**d,e)**: n = 2 in triplicate; one-way ANOVA. Tukey post hoc; *p* < 0.05. * vs. C; # vs L; ɸ vs shATG5/leptin.

Cell migration is considered as a highly energy-demanding process because the cell deals with mechanical and signaling changes in the organization of the cytoskeleton and the extracellular matrix to achieve cell motility^30^. In a previous work, we demonstrated that autophagy participates in leptin-induced migration in breast cancer cells^19^. Since we show that autophagy is required for ATP production and leptin-enhanced mitochondrial fitness in breast cancer cells (Figs. 3 and 4), we hypothesized that autophagy and mitochondrial metabolism could be involved in leptin-induced cell migration. Accordingly, we measured changes in cellular migration in highly invasive breast cancer cells (MDA-MB-231) which had high ATP production by glycolysis (Fig. 3d), treated with leptin, CQ, or oxidative phosphorylation (OXPHOS) inhibitors that prevent mitochondrial ATP formation (oligomycin and antimycin A). The data showed that in control conditions, OXPHOS inhibition reduced cell migration at 24 h (Fig. 5a), while at 48 h, this effect was not maintained (Fig. 5b), and cells migrated similarly to the control (Fig. 5a,b, Supplementary Fig. S8). In the presence of leptin, cell migration increased significantly at 24 and 48 h. Leptin-induced cell migration was prevented by autophagy inhibition with CQ at both time points evaluated. Also, OXPHOS inhibition significantly reduced leptin-induced migration, but to a lesser extent than the inhibition of autophagy (Fig. 5a,b, Supplementary Fig. S8). Similar data was obtained in poorly invasive MDA-MB-468 cells with high mitochondrial ATP production (Fig. 3c, Supplementary Fig. S9), indicating that autophagy and ATP production are required to sustain leptin-induced migration in cells with high or low invasive capacity. Since leptin induced cell invasion which is a fundamental step in the metastatic cascade^31^, we explored the role of autophagy and mitochondrial ATP in the invasion of leptin-treated MDA-MB-231 cells. As expected, leptin increased the invasiveness of MDA-MB-231 cells (Fig. 5c). Importantly, inhibition of autophagy with CQ and inhibition of ATP production reduced leptin-induced invasion. This data suggests that autophagy and mitochondrial ATP production are necessary for leptin-induced migration and invasion.

To demonstrate that the results are a consequence of deficient autophagy and not CQ unspecific effects, we replicated the migration assay in MDA-MB-231 cells with deficient autophagy due to ATG5 knockdown (Fig. 4e). The data showed that in the absence of leptin, autophagy inhibition decreased cellular migration at 24 and 48 h, but migration was further decreased with the use of mitochondrial inhibitors oligomycin and antimycin A (Fig. 5d,e, Supplementary Fig. S10). In agreement with the previous result, leptin increased the migration in non-silencing cells, and OXPHOS inhibitors reversed this effect (Fig. 5d,e, Supplementary Fig. S10). In ATG5 knockdown cells, leptin did not increase cell migration at both time points evaluated. Additionally, in ATG5 knockdown cells treated with leptin, the use of OXPHOS inhibitors further reduced cell migration. These data suggest that autophagy and mitochondrial metabolism are required to sustain leptin-induced migration in invasive breast cancer cells, and also suggests that a full inhibition of OXPHOS is needed to completely avoid migration in these cells.

## Discussion

Autophagy is one of the main processes of degradation and recycling of intracellular components, maintaining homeostasis, and allowing cell adaptation and survival in adverse physiological conditions such as starvation or hypoxia^9^. In cancer, the role of autophagy has been widely explored, and it is recognized that in the early stages prevents malignant transformation. Still, in an established tumor, it is necessary for proliferation, acquisition of metastatic potential and increased tumor malignancy^8,9,32,33^. Since proliferation and survival of some cancer cells driven by RAS/MAPK oncogenic mutations were identified as addicted to the autophagic process, targeting autophagy has been implemented as a potential therapeutic strategy^10,22,34^. However, the outcome of this therapeutic approach in cancer clinical trials has not been conclusive and presumably depends on the tumor context^8^.

Since leptin was shown to induce autophagy in healthy peripheral tissues, the interest has grown in elucidating the role of autophagy in leptin-driven malignant features of obesity-linked cancer^26^. Postmenopausal breast cancer is one of the malignancies associated with obesity in women. In obesity, the secretion of adipokines, cytokines, and hormones that have been associated with carcinogenesis and tumor progression, is altered^35^. Among all the bioactive molecules deregulated in obesity is the adipokine leptin. According to the evidence, in obese breast cancer patients, high leptin levels in plasma and overexpression of leptin receptors in tumors correlate positively with poor prognosis^36,37^; and mice genetically deficient for the leptin receptor do not develop oncogene-induced mammary tumors, as a result of defective leptin signaling^38^. In in vitro models of breast cancer, the role of leptin on proliferation, migration, epithelial-mesenchymal transition (EMT), and cell invasion by activating signaling pathways such as ERK, STAT3, and FAK-Src has been described^31,39,40^. In this work, we used cell lines that represent two breast cancer subtypes: the hormone receptor-positive subtype and triple-negative subtype. Similar to other reports, we demonstrate that leptin promoted tumor characteristics of malignancy such as mesenchymal-like cell morphology (Fig. 1e) and increased proliferation in hormone receptor-positive cells (Fig. 1a,b) but not in triple-negative cells (Fig. 1c,d). Also, our data showed that leptin increased ERK activation (Fig. 1f–i), a major signaling pathway linked to proliferation^41^. These data demonstrate that leptin favors cancer cell aggressiveness and, since leptin did not increase pAKT and pJAK in all cancer cell lines (Supplementary Fig. S2), increased ERK activation in all cell lines and only increased proliferation in ER^+^/PR^+^ cells, it suggests an alternative mechanism to the ERK pathway by which leptin increases proliferation in hormone receptor-positive cells and not triple-negative breast cancer cells. Alternatively, ERK could participate in leptin-induced proliferation in ER^+^/PR^+^ cells with low basal levels of ERK activation and not in triple-negative cells with higher levels of basal ERK activation. High basal ERK levels in triple-negative cells could be associated with their high proliferation or invasive capacity and increased ERK phosphorylation induced by leptin could have effects unrelated to proliferation.

We have reported previously that leptin induces autophagy in ER^+^/PR^+^ cells but not in triple-negative cells^19^. Our present results are consistent with the former study (Fig. 2a,b) and demonstrate that the inhibition of leptin-induced autophagy impairs proliferation increased by leptin in hormone receptor-positive cells (Fig. 2c,d), indicating the participation of autophagy in cellular proliferation induced by leptin. Although leptin did not induce autophagy (Fig. 2b) or proliferation in triple-negative cells (Fig. 1c,d), both cell lines responded differently to basal autophagy inhibition (Fig. 2e,f). In MDA-MB-468 cells, autophagy inhibition did not affect proliferation, but it did in MDA-MB-231 cells (Fig. 2e,f). This effect is controversial since it has been described that triple-negative cells in basal conditions are dependent on autophagy for survival because they have alterations in pathways related to autophagy-dependency such as RAS/MAPK pathway, p53, or activation of STAT3 or EGFR pathways^8,42^. Therefore, we do not discard that the lack of sensitivity to the inhibition of autophagy observed in the MDA-MB-468 triple-negative cells may be related to the experimental conditions used in our study since cells were serum-deprived before leptin treatment. In this case, additional stress signals could be activated in this particular cell line allowing survival to CQ treatment. Overall, we suggest that the biological effect of leptin on hormone receptor-positive breast cancer cell proliferation is mediated by autophagy. Although leptin eventually does not modify autophagy or proliferation of triple-negative cells, basal levels of autophagy active in these cells could participate in other leptin-induced tumor characteristics, as we will describe regarding metabolism and cellular migration.

Metabolic reprogramming is a hallmark of cancer associated with cellular elevated proliferation rate^43^. In most solid tumors, the Warburg effect, or aerobic glycolysis, characterized by high glucose uptake and lactate release, has been recognized as the dominant metabolic profile that sustains macromolecule biosynthesis and energy production^43^. In some mammary tumors, a glycolytic metabolic profile has been described^44^. However, although glycolysis might be increased in cancer cells, it has also been suggested that mammary tumor cells increase their mitochondrial activity to promote metastatic colonization in distant organs^45–49^. In this regard, some authors propose that enhanced mitochondrial respiration during cancer progression towards metastasis provides abundant ATP to drive migration and colonization of distant sites^50^. Importantly, the tumor microenvironment has a key role in this metabolic reprogramming, either providing energetic substrates or secreting signaling molecules to support the metastasis cascade and reactivating cellular proliferation in the metastatic target organ. For example, in the metastatic site, nutrients and oxygen are not usually limiting, unlike the primary tumor. So, the increased availability of nutrients and oxygen at the metastatic site together with increased mitochondrial respiration is likely an efficient way to meet the energetic needs associated with metastasis establishment^50^. In obesity-associated cancer, when metastatic breast cancer cells are cultured with serum from obese mice, changes in the expression of genes associated with metabolism occur, including fatty acid metabolism, mTOR signaling, and autophagy^50^, indicating an important role for adipocyte signaling in tumor cell metabolism. Particularly, the increase in leptin secretion mediated by adipocytes can influence tumor cell metabolism, and also the metabolism of cells adjacent to the tumor^51^. For example, it has been shown that leptin influenced the protein cargo and the biological activity of extracellular vesicles secreted from breast cancer cells, increased mitochondrial metabolism of breast cancer cells, and also regulated macrophage metabolism, which are the most abundant immunological component in the tumor microenvironment^52^. Additionally, it has been demonstrated in estrogen receptor-positive (ER^+^) cells, that leptin increased mitochondrial ATP production and enhanced mitochondrial respiration^53–55^. On the other hand, in pancreatic cancer cells, leptin was reported to increase glucose uptake, lactate production, and expression of the glycolytic enzyme hexokinase II (HK II)^56^. Our data agree with the previous evidence and demonstrates that leptin not only enhances mitochondrial function and ATP production in hormone receptor-positive cells (Fig. 3a,b) but also in triple-negative breast cancer cells (Fig. 3c,d).

Additionally, we demonstrate for the first time that leptin increased glycolytic ATP production in triple-negative breast cancer cells (Fig. 3c,d). Although it has been suggested that the effect of leptin on cellular metabolism could be mediated by increased mitochondrial biogenesis and dynamics^54^, recently Pham et al. revealed that leptin-induced increase in β-oxidation and OXPHOS activity are potentially regulated by autophagy^57^. Interestingly, our results revealed that pharmacological and genetic inhibition of autophagy prevented the leptin-induced enhancement in mitochondrial function in hormone receptor-positive and triple-negative cancer cells (Figs. 3a–d, 4a–g, Supplementary Fig. S5–S7) suggesting a general effect of autophagy in mediating mitochondrial function in breast cancer cells. Also, we show that autophagy inhibition reverted the leptin-induced increase in glycolytic ATP production in triple-negative breast cancer cells (Fig. 3c,d), indicating that autophagy also supports glycolysis in this breast cancer subtype.

Although we did not examine the molecular mechanism by which autophagy regulates leptin-driven glycolytic and mitochondrial metabolism, Pham et al. described in ER^+^ cells that autophagy induces SREBP-1, a transcription factor which mediates leptin-induced fatty acid metabolism increasing mitochondrial ATP production^57^. This mechanism represents a potential pathway by which autophagy regulates leptin-induced metabolism. However, multiple regulatory pathways could be involved. For example, concerning mitochondrial metabolism, it has been described that the degradation of mitochondria by autophagy promotes healthy mitochondria and preserves mitochondrial integrity and function^58^. Additionally, the degradation of cytoplasmic components by autophagy can provide metabolites that sustain the tricarboxylic acid cycle (TCA)^18^.

Regarding glycolysis regulation, the degradation of glycolytic enzymes, such as HK II, by autophagy can disrupt the glycolytic process and eventually allow domination of the mitochondrial function by anaplerosis^59^. Nevertheless, this last option seems to be unlikely since we did not see a decrease in glycolytic ATP production upon leptin treatment and it even increased in triple negative cells. Autophagy has also been shown to support RAS-driven glycolytic activity^60^. Additionally, it has been revealed that autophagy regulates the glycolytic profile by increasing Monocarboxylate Transporter 1 (MCT1) expression and increasing lactate release through a mechanism involving activation of the Wnt/β-catenin pathway^61^. According to the authors, increased glycolysis induced by autophagy was associated with metastasis of hepatocellular carcinoma cells^61^. As a product of glycolysis, the lactate released by tumor cells in the extracellular space is essential for the metastatic process allowing acidification of the tumor microenvironment, which is favorable for the activation of proteases such as matrix metalloproteinases (MMPs), urokinase-type plasminogen activator, and cathepsins B, D, and L inducing the degradation of the extracellular matrix (ECM) and facilitating tumor cell metastasis^62^.

In general, we suggest that leptin-induced metabolic changes are required to sustain features of increased tumor aggressiveness associated with metastasis. In this scenario, autophagy is an easily-activated process that the cell uses to speed up nutrient bioavailability and satisfy the energetic demands required to sustain cancer features considered of a high energetic demand. Moreover, metabolic changes regulated by autophagy could influence the metabolism of non-tumor cells adjacent to the tumor, since it was suggested that leptin induced extracellular vesicle secretion with an important role in the metabolic crosstalk within the immune cells in the breast tumor microenvironment in which leptin concentration could be higher than in the blood^52^. This hypothesis needs to be tested in future studies.

Throughout the invasion-metastasis cascade, tumor cells are exposed to multiple factors that challenge their survival. In this process, one of the initial steps consists of local invasion of the basement membrane and cell migration^63^. Both events need energy distributed among the organization and contractility of the cytoskeleton, the extension of invadopodia which apply a combination of protrusive and contractile forces to physically open spaces in the matrix and move through them, and even for the transport of MMPs towards the invasive front to degrade the ECM^30^. In this way, modulation of metabolism in cancer cells is a strategy that allows the acquisition of increased invasive properties. Evidence suggests that migratory cells use mitochondrial function to improve ATP production and glycolysis to modify the microenvironment in the ECM and permit migration^14,30^. In this regard, we demonstrate that leptin drives mitochondrial function and glycolytic ATP production in triple-negative breast cancer cells (Fig. 3c,d) with different migration capacities and invasive potential^64^, and more importantly, we reveal that autophagy is a mechanism that regulates leptin-induced metabolic changes responsible for the migratory and invasive potential (Figs. 3c,d, 4c,d,g, 5, Supplementary Fig. S9).

In our previous work, we have revealed that inhibition of autophagy reverted leptin-induced migration of MDA-MB-231 cells^19^. Our data support the above since leptin-increased cell migration in triple-negative cells and pharmacological and genetic inhibition of autophagy reduced the effect of leptin (Fig. 5a,b,d,e, Supplementary Fig. S8–S11). Additionally, the results suggest that autophagy also regulates leptin-induced cell invasion (Fig. 5c). Although a previous study has proposed that leptin regulates cell migration and invasion through several mechanisms such as FAK-Src, TGFβ1 axis, and IL-18 pathway^24,31,65^, we demonstrate that the regulation of metabolism by autophagy supports leptin-induced cell migration and invasion (Figs. 4,5, Supplementary Fig. S9). More importantly, our data shows that autophagy inhibition was more efficient in reducing leptin-induced cell migration than OXPHOS inhibition (Fig. 5a,b,d,e, Supplementary Fig. S8–S10). This could be because autophagy inhibition reduced both the leptin-mediated increase in mitochondrial function and glycolytic ATP production (Figs. 3c,d, 4c,d,g), decreasing both ATP sources and leaving the cell with less energy available for cell migration. Moreover, autophagy could also be regulating other mechanisms that regulate migration and invasiveness, as has been demonstrated for focal adhesion turnover^66^.

Importantly, the knockdown of the autophagy gene ATG5, combined with OXPHOS inhibitors, completely suppressed leptin-induced migratory ability and basal migration (Fig. 5d,e, Supplementary Fig. S10). These results suggest that basal metabolic processes in cancer cells still support cancer cell migration even in the instance of autophagy inhibition and the consequent prevention of glycolytic and mitochondrial metabolic changes induced by leptin. Moreover, our data indicates that the inhibition of autophagy in combination with OXPHOS impairment, could be an efficient strategy to stop metastasis-associated processes such as leptin-induced cell migration. Although our data are focused on the effect of leptin, we do not discard that the combined inhibition of autophagy and OXPHOS could be an appropriate strategy in triple-negative mammary tumors, which have shown high expression of autophagy markers, that positively correlate with poor prognosis, and which also show enrichment of pathways associated with OXPHOS in the metastatic stage, despite being tumors that originally depend on a glycolytic metabolism^44,48,67^, particularly in the setting of obesity.

Similar to the promising therapeutic approach of autophagy modulation, increased mitochondrial metabolism is currently considered a metabolic vulnerability of cancer cells during metastasis^68^ and several studies support this proposal. For instance, in order to survive in the blood or lymphatic vessels, circulating cancer cells enhanced OXPHOS and increased ATP production through PGC-1α and mitochondrial biogenesis^47^. Also, it has been shown that mitochondria in cancer cells can utilize a broad range of metabolic pathways such as glucose oxidation, fatty acid β-oxidation (FAO) and glutamine oxidation to fuel the electron transport chain (ETC) for ATP production^69^. This metabolic flexibility in cancer cells allows chemotherapy resistance, and supporting tumor recurrence and metastatic progression^68,70^. Interestingly, in metastatic tumors, mitochondrial gene expression is increased, and corelated with a worse overall survival and poor prognosis in patients^71–73^. Because of the importance of mitochondrial function in metastasis, strategies targeting the TCA and OXPHOS have been suggested, and in vitro models result in promising outcomes but achieve only modest benefits in clinics^13,74^. These effects are due, in part because in preclinical models, the concentration of inhibitors is extremely high and could represent toxic effects at the clinical level, in addition to exceeding concentrations allowed by the FDA for clinical studies^13^. In general, combining other therapeutic strategies, such as chemotherapy with autophagy inhibition or mitochondrial metabolism, has shown better outcomes than individual administration^69,75^. So, we describe a possibility to modulate cancer cell metabolism by regulating autophagy or to prevent leptin-induced cell migration by autophagy and/or OXPHOS inhibition in triple-negative breast cancer cells.

Obesity represents an important risk factor for breast cancer, affecting its incidence, prognosis and the mortality rates particularly in relation to the menopausal status. Obesity is also associated with a reduced effectiveness of chemotherapeutic agents, and an increased risk of complications associated with surgery and radiation^76^. An important molecular link between obesity and antitumor resistance is increased leptin secretion, which can affect the intrinsic molecular characteristics of breast cancer cells and influence the therapeutic response of patients^76^. Due to the role of leptin-mediated signaling in obesity-link cancer, several therapeutic agents have been designed to modulate the leptin cascade, such as leptin receptor and leptin antagonists^35^. However, the effect of this therapeutic approach has only been tested in preclinical studies with attractive outcomes, but clinical trials have not been conducted in breast cancer patients^35^. In this context, our data showed that leptin induced metabolic changes regulated by autophagy that are associated with increased cell migration and invasive capacity. We do not discard that these changes could be a mechanism by which leptin confers therapeutic resistance, especially because the literature strongly supports the role of autophagy and metabolism in resistance mechanisms of multiple cancers^77^. So, targeting autophagy in combination with therapy in order to block cancer cell metabolic reprogramming might represent a possible strategy to overcome therapy resistance in obese patients. However, the optimal use of antitumoral treatments, especially in targeting autophagy, in women with obesity needs to be further evaluated, since the data about therapeutic regimens, body mass index, and outcomes are not conclusive, and in some cases autophagy inhibition could promote tumorigenic characteristics such as EMT^8^.

In conclusion, in this study, we demonstrated that autophagy regulates different biological responses induced by leptin. In ER^+^/PR^+^ cancer cells, leptin increased autophagy, promoting proliferation and leptin-enhanced mitochondrial metabolism, possibly for maintaining proliferation and cell migration. Additionally, in triple-negative breast cancer cells, autophagy regulated mitochondrial metabolism, glycolytic ATP production and leptin-enhanced cell migration and invasion. For the first time, our data demonstrates that inhibition of autophagy and OXPHOS can avoid the migratory capacity of highly invasive cells exposed to leptin and contributes to underscoring the importance of metabolic changes and their modulators, like leptin or autophagy, in obesity-related cancer.

## Methods

### Reagents

Eagle’s Minimum Essential Medium (Eagle’s MEM, Sigma-Aldrich, M0268); Dulbecco’s Modified Eagle’s Medium/Nutrient blend F-12 Ham (DMEM/F12, Caisson, DFP18); fetal bovine serum (FBS, Biowest, BIO-S1650); RPMI (Caisson, 1640); insulin (Sigma-Aldrich, I0516); penicillin/streptomycin (P/S, Caisson, PSL01); puromycin (Sigma-Aldrich, P8833); human recombinant leptin (Sigma-Aldrich, L4146); Chloroquine diphosphate salt (CQ, Sigma-Aldrich, C6628); propidium iodide (PI, Sigma-Aldrich, P4170); polybrene (hexadimetridine bromide, Sigma-Aldrich, 107689); TransIT-LT1 (Mirus, MIR2300) Seahorse XF DMEM Medium, pH 7.4 (Agilent Technologies, 103575-100); Seahorse XF glucose solution (Agilent Technologies, 103577-100); Seahorse XF sodium pyruvate solution (Agilent Technologies, 103578-100); Seahorse XF L-glutamine solution (Agilent Technologies, 103579-100); Seahorse XF calibrant solution (Agilent Technologies, 100840-000); Seahorse XF Real-Time ATP Rate Assay Kit: oligomycin, rotenone/antimycin A (Agilent, 103591-100); Seahorse XF Mito stress test kit: oligomycin, FCCP (Carbonyl cyanide-4 (trifluoromethoxy) phenylhydrazone), rotenone/antimycin A (Agilent Technologies, 103015-100).

### Cell culture

Two cell lines ER^+^/PR^+^ were used under the following conditions: MCF-7 cells were cultured in Eagle´s MEM supplemented with 10% FBS, 10 µg/mL insulin, and Penicillin (100 U/mL)/ Streptomycin (100 µg/mL) (P/S). T-47D cells were grown in RPMI with 10% FBS, 7.5 µg/mL insulin, and P/S. Two triple-negative cell lines, MDA-MB-468 and MDA-MB-231, were cultured in DMEM/F12 with 10% FBS and P/S. All cell lines were maintained under a 5% CO2 atmosphere at 37 °C. Cell lines were a kind gift from Dr. Andrew Thorburn at the University of Colorado, Denver. They were initially acquired from the Tissue Culture Core at the University of Colorado where they were fingerprinted and known to match ATCC cell lines. Cells were thawed and were not grown for longer than 6 months in order to avoid culture variability.

### Leptin, chloroquine, oligomycin, and antimycin A stimulation

For all assays, cells were previously synchronized by serum deprivation for 24 h. Next, ER^+^/PR^+^ cell lines were treated with 400 ng/mL and triple-negative cell lines with 50 ng/mL of human recombinant leptin dissolved in 0.01% bovine serum albumin for leptin treatment. CQ was employed at 20 µM for all cell lines. For autophagy flux, cells were treated with the indicated leptin concentrations for 24 h in serum-free media. Two hours before protein extraction, CQ was added to allow autophagosome accumulation and to evaluate changes in the LC3 II autophagy marker. For proliferation and metabolic assays, leptin (L), and/or CQ were added at time 0 in serum-free media and were maintained for 24 or 48 h until the corresponding experiment was performed. For the wound-healing assay, oligomycin was used at 0.5 µM and antimycin A at 5 µM. Both were used during 24 h or 48 h. The control was treated with bovine serum albumin 0.01% which is the leptin vehicle.

### Western blot

Total proteins were obtained by lysis using RIPA buffer with protease inhibitors (Complete protease inhibitor cocktail, Sigma, 11697498001) and phosphatase inhibitors (PhosSTOP, Sigma, 4906845001). Proteins were quantified by Bradford’s method, and 30 µg were resolved by 10–15% SDS-PAGE and electrotransferred (semi-wet transfer, 15 V 50 min) to polyvinylidene difluoride (PVDF) membranes. Membranes were blocked with 5% skim milk in TBS-Tween buffer for 1 h at room temperature and then incubated overnight at 4 °C with primary antibodies: anti-JAK2 (1:1000, ABclonal, A11497), anti-phosphorylated JAK2 (Y1007/1008) (1:1000, ABclonal, AP0531), anti-AKT (1:1000, Cell Signaling, 9272), anti-phosphorylated AKT (Thr308) (1:1000, Cell Signaling, 9274), anti-ERK (1:1000, Cell Signaling, 4695), anti-phosphorylated ERK (1:1000, Cell Signaling, 4370), anti-LC3B (1:2500, Novus, NB100-2220), anti-ATG5 (Cell signaling, 2630) or anti-β-actin (1:5000, Sigma-Aldrich, A5441). Later, membranes were incubated for 1 h at room temperature with secondary HRP-linked anti-mouse IgG (1:20,000, Sigma-Aldrich, A2304) or anti-rabbit IgG-Peroxidase (1:20,000, Sigma- Aldrich, A0545) antibodies. Finally, immunodetection was performed with HRP-Western chemiluminescent Immobilon Substrate (Millipore, WBKLS0500) on the C-DiGit transfer scanner (LI-COR Biosciences). The relative intensity of bands was obtained by densitometric analysis using Image J software. Complete blots for all the membranes presented in the figures are included in Supplementary Figs. S12–S16. For LC3 Western Blots, membranes were cut prior to hybridization with primary antibodies to separate LC3 (16/14 kD) and actin (42 kD) bands. We have previously performed extensive validation of these antibodies with appropriate controls to identify both bands.

### Proliferation and cell death analysis

The proliferation analysis was monitored in a real-time IncuCyte Zoom microscopy system (Zoom GUI analysis software v2015A; Zoom controller v2015A www.essenbioscience.com), and graphed as percent cellular confluence for 48 h. 8 × 10^3^ MCF-7, MDA-MB-468, MDA-MB-231 cells, and 6 × 10^3^ T-47D were plated in 96 multi-well plates. Once adhered, cells were treated with leptin or CQ in triplicate. After 48 h of treatment, cell death was measured by propidium iodide (PI) staining (1 μM) for 10 min, and red fluorescence was evaluated in the IncuCyte ZOOM system. Cell death was expressed as the percent confluency of red fluorescence normalized to the total percent confluency calculated by the IncuCyte ZOOM analysis software. For the carboxyfluorescein succinimidyl ester (CFSE) assay, the CFSE Cell Division Tracker Kit (Biolegend, 423801) was used. Briefly 2 × 10^5^ cells/ well were plated in six-well plates per condition. After adherence, all cells were serum deprivation for 24 h, and staining was carried out with 1 µM CFSE and cells were incubated at 37 °C for 30 min. Next, the treatments were placed for 24 and 48 h. At the end of each treatment, cells were trypsinized and centrifuged at 2500 rpm. The pellet was resuspended in 1X PBS with 3% FBS. Filtered samples to avoid cell clustering were read using a BD FACS Canto flow cytometer. The data were processed with Flow Jo v10.10 software (www.flowjo.com).

### shRNA lentiviral transduction

For genetic inhibition of autophagy, pLKO.1 shRNA vector was used in a lentiviral system. Lentivirus production was performed by transfection of 1.8 × 10^6^ LentiX-293 T cells (Clontech, NC983969) with TransIT-LT1 transfection reagent, packaging plasmids (0.2 μg pVSV-G, 1.8 μg pRSV and 1.8 μg pRRE) and 2 µg pLKO plasmid (non-silencing sequences, NS or ATG5, TRCN0000151474). Lentiviral media were collected at 24 and 48 h, spiked with 0.8 μg/mL polybrene (hexadimethrine bromide, Sigma, 107689), and stored at –80 °C. For transduction, 1.8 × 10^6^ MCF-7 cells were seeded in a six-well plate and, after adherence, were incubated for 1 h with polybrene (8 μg/mL) and for 24 h with 1 mL of viral media. After 24 h, the selection process was started with puromycin (0.5 μg/mL) for 3 days. Selected cells were maintained with low doses of puromycin (0.3 μg/mL) to allow proliferation. Cells were used after a single passage to avoid loss of shRNA silencing.

### Metabolic profiling by Seahorse XFe96

The following cell densities were seeded in XF96 culture microplates (Agilent 101,085–004) per well: 2.5 × 10^4^ MCF-7 and MDA-MB-468; 1.5 × 10^4^ T-47D and MDA-MB-231. After adherence and synchronization by serum deprivation, treatments with leptin and/or CQ were applied for 24 h. Next, the cell culture medium was replaced by DMEM XFe Medium, pH 7.4 supplemented with 10 mM of glucose, 1 mM of pyruvate, 2 mM of glutamine, and were incubated for 45 min at 37 °C in a non-CO_2_ atmosphere. The medium was replaced with pre-warmed DMEM XFe Medium, pH 7.4 (supplemented), and cells were incubated at 37 °C in a non-CO_2_ atmosphere until the metabolic measuring. Reagents were loaded into the sensor cartridge as indicated by the manufacturer. The Real-Time ATP Rate Assay Kit was used, with the following reagent concentrations: oligomycin (2.5 μM), rotenone/ antimycin A (0.5 μM each). For the Mito stress test kit, oligomycin (2.5 μM), FCCP (1 μM) and rotenone/antimycin A (0.5 μM each) were used for the analysis of mitochondrial function. For viral transduced cells with shATG5 or NS, the same strategy was used, but FCCP was used at 0.5 μM since this was the lowest FCCP concentration at which the cells reached their maximal respiration. For all experiments, the equipment was calibrated following the manufacturer's specifications and after completing the calibration, the OCR and ECAR were measured before and after the sequential injection of the reagents of each metabolic assay kit. ATP production and mitochondrial parameters were calculated using the Agilent Seahorse Wave Controller 2.6 software according to the manufacturer´s instructions. All the data obtained was normalized to cell number.

### Wound healing assay

To measure cell migration, 1.5 × 10^3^ MDA-MB-231 cells (normal, shATG5 or NS) or 3 × 10^4^ MDA-MB-468 cells were seeded per well in 96-well plates. After adherence and synchronization by serum deprivation, the wound was made using an Essen Bioscience Wound Maker. Next, cells were washed twice with PBS to remove unattached cells. Treatment was applied, and cell migration was monitored at 24 h and 48 h. Images were obtained by EVOS M7000 Thermo microscope (Thermo Fisher Scientific) using a 4 × objective. In all images, the wound healing was analyzed using Image j v1.50i (https://imagej.net/ij/ij/). The data obtained is presented as a percent wound healing area.

### Cell invasion assay

Matrigel invasion assay was carried out using 24-well Transwell inserts (Corning, 354578) coated with 100 µL of Matrigel (250 µg/mL; Corning, 35434) pre-incubated at 37 °C for 30 min. Previously, MDA-MB-231 cells were pre-treated with leptin, chloroquine and oligomycin during 48 h. After, cells were recovered and 5 × 10^4^ cells per treatment were resuspended and seeded in 50 µL of serum-free medium into the upper Transwell insert. 750 µL of supplemented medium (DMEM/F12 with 10% FBS) were placed in the lower chamber. Cells were incubated for 48 h at 37 °C in a 5% CO_2_ atmosphere. Following incubation, cells and Matrigel on the upper surface of the Transwell membrane were gently removed with cotton swabs. Cells invading through the membrane were washed and fixed with formaldehyde (3.7% in PBS) for 10 min and next with methanol for 10 min. After, fixed invading cells were stained with 0.4% crystal violet. Finally, five images of the invasive cells by condition were obtained by EVOS M7000 Thermo microscope (Thermo Fisher Scientific) with the 20× objective microscopy. Quantification was performed using Image jv1.50i (http://imagej.net/ij/ij/), measuring the area occupied by cells. Data are presented as percentage of invaded area.

## Statistical analysis

Graphs and statistical analysis were performed using GraphPad Prism v6.01. (www.graphpad.com) software. All data were shown as mean ± standard deviation (SD) of three or more independent experiments unless stated otherwise. Statistical analysis was performed using one-way or two-way ANOVA, with Tukey, Dunnett, or Sidak post-hoc test. *P* values < 0.05 were considered statistically significant.

### Supplementary Information

Supplementary Figures.

## Data Availability

All data generated or analysed during this study are included in this published article [and its supplementary information files].
