# Supplementary material for: Regulation of mitochondrial metabolism by autophagy supports leptin-induced cell migration
Source: Sci Rep. 2024 Jan 16;14:1408. doi: 10.1038/s41598-024-51406-y (PMC10791685; doi:10.1038/s41598-024-51406-y)
Supplement: Supplementary file 1 — Supplementary Figures. [file 41598_2024_51406_MOESM1_ESM.docx]

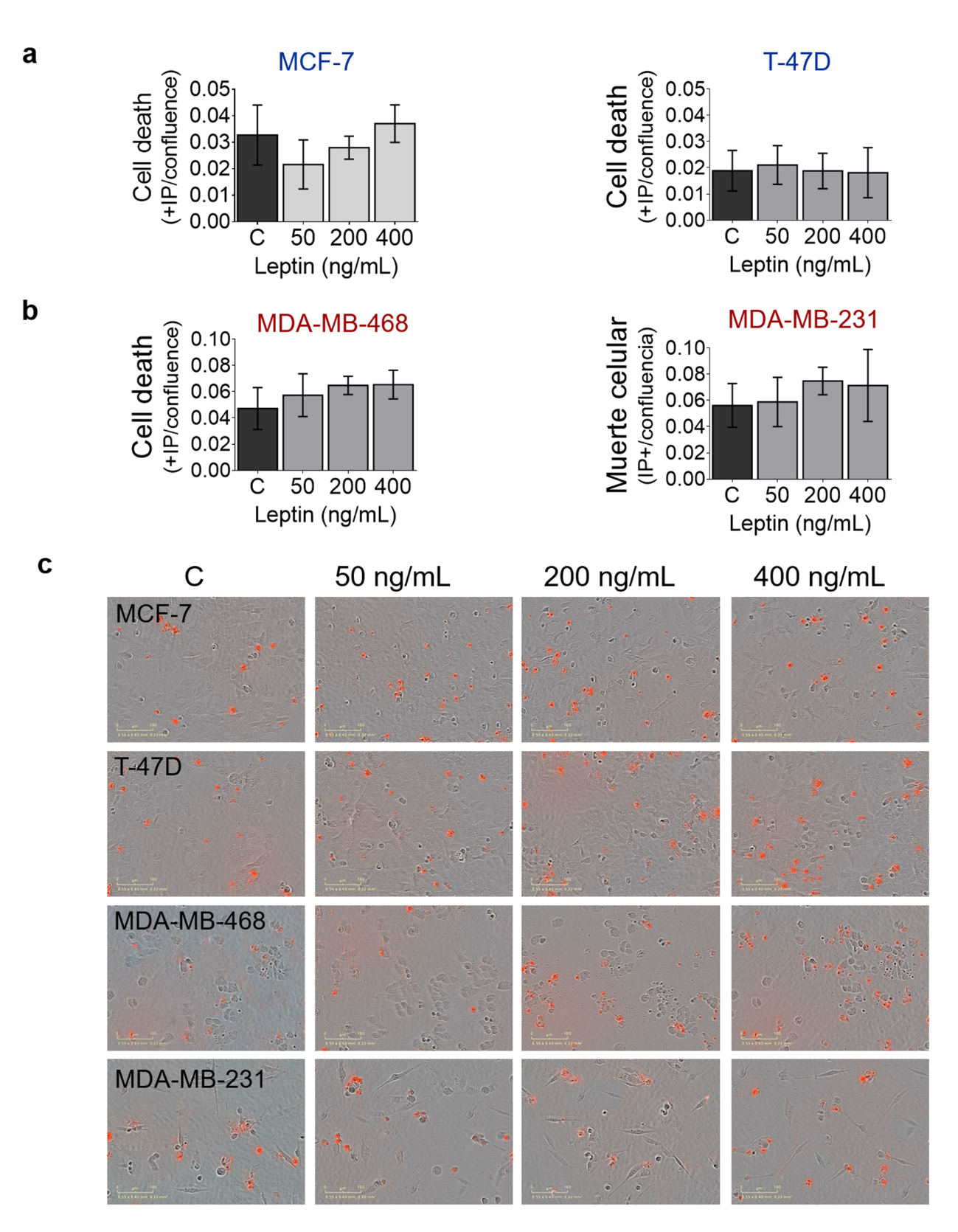


**Supplementary Figure S1. Effect of different leptin concentrations on breast cancer cell death.** All cells were treated with different concentrations of leptin for 48 h. After leptin treatment, cells were incubated in a 10 μM propidium iodide (PI) solution for cell death assessment. Leptin treatment did not induce cell death in the breast cancer cell lines studied (a-b). In the representative images no differences were observed in the number of PI-positive cells (c). The control was treated with a vehicle. C: control. mean ± S.D.; n=2 in quadruplicate. One-way ANOVA; Tukey post hoc. No significant differences were found.


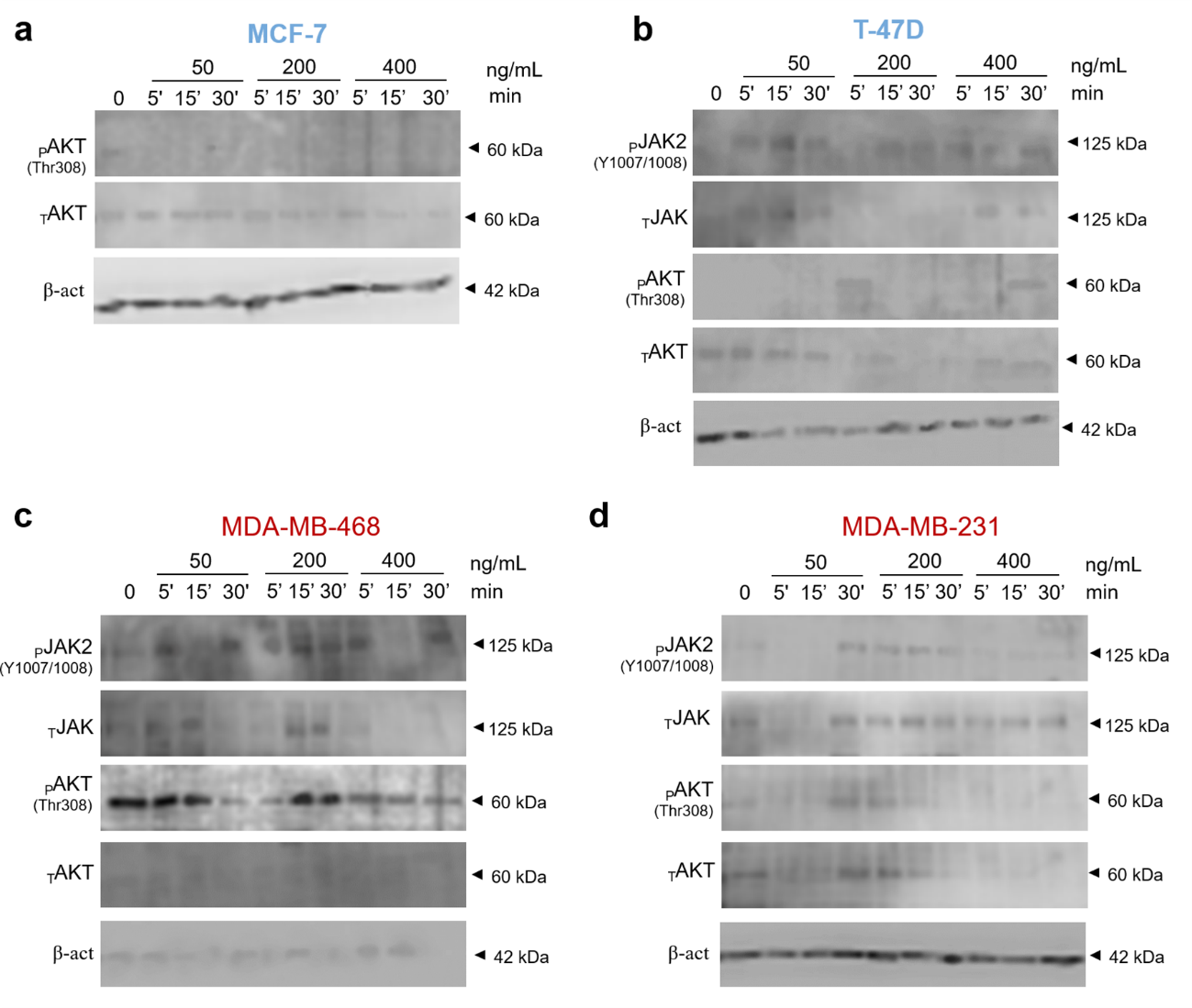


**Supplementary Figure S2. Effect of leptin on AKT and JAK phosphorylation in breast cancer cells.** Cells were treated with 50, 200 and 400 ng/mL of leptin during 5, 15 and 30 min. Leptin induced JAK activation (Y1007/1008) and AKT phosphorylation (Thr 308) in T-47D, MDA-MB-468 and MDA-MB-231, but not in MCF-7 cells. In T-47D cells, the highest activation of JAK was observed at 50 ng/mL of leptin (b), While in MDA-MB-231 pJAK was highest at 50 and 200 ng/mL of leptin (d). Importantly, in MDA-MB-468 JAK activation was similar in all leptin concentrations (c). On the other hand, 200 and 400 ng/mL of leptin induced pAKT in T-47D cell (b), whereas, in triple-negative cells pAKT was highest at 50 and 200 ng/mL of leptin (c, d). phospho-JAK, total-JAK and phosphor-AKT were not detected under experimental conditions in MCF7 cells (a). The original blots are available in Supplementary Figure S16.


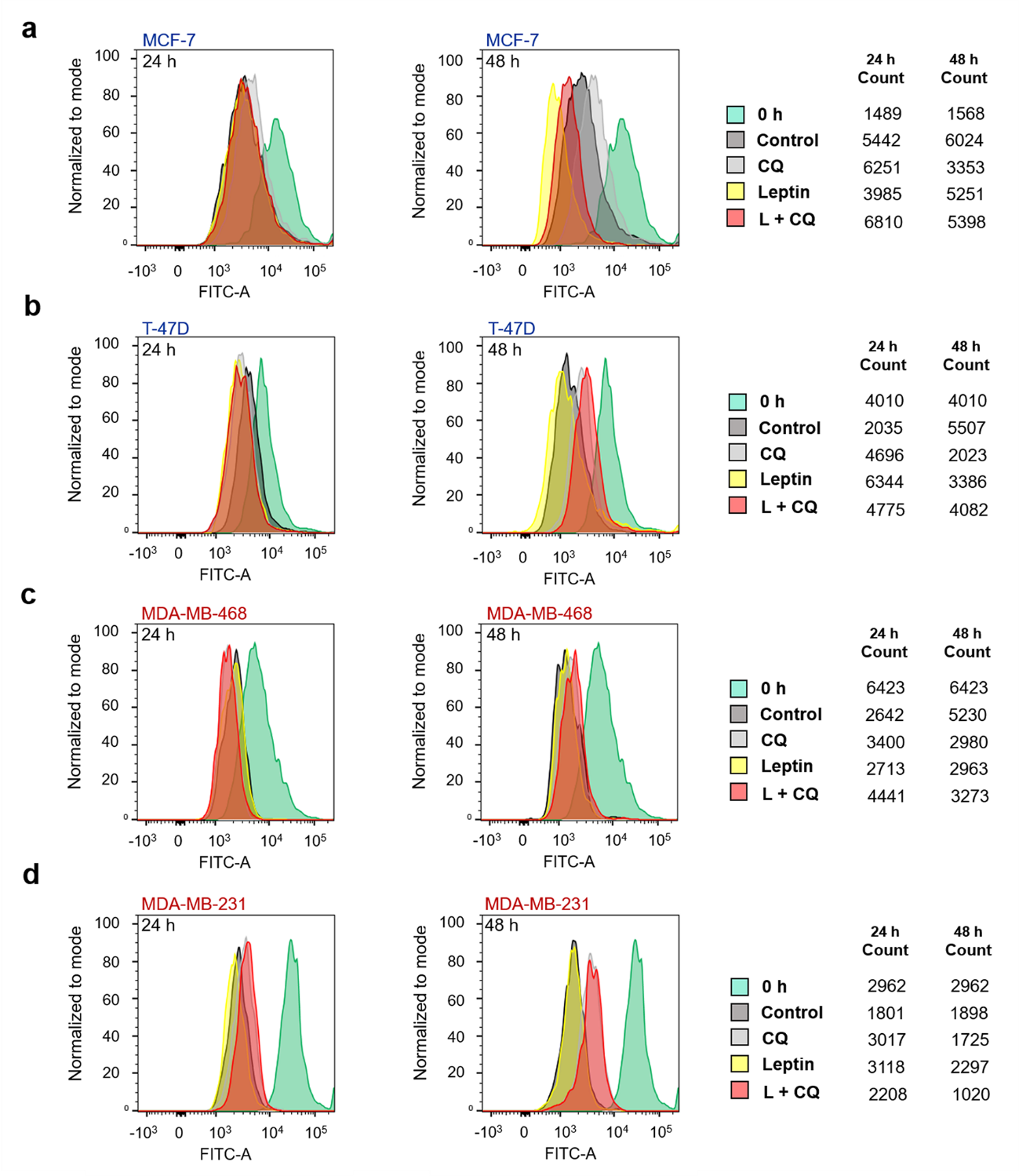


**Supplementary Figure S3. Effect of autophagy on cell proliferation of breast cancer cells treated with leptin.** Cell proliferation was measured by CFSE assay. Cells were stained with CFSE at 1µM during 30 min at 37 °C. After, CFSE-labeled ER^+^/PR^+^ cells and CFSE-labeled triple-negative cells were treated with 400 ng/mL and 50 ng/mL of leptin respectively, and/or chloroquine at 20 µM for 24 and 48 h. Cells were analyzed using flow cytometry. Proliferation was indicated by a decrease in fluorescence intensity. Leptin increased cell proliferation at 48 h in ER^+^/PR^+^ compared to the control (a, b). Interestingly, autophagy inhibition reduced leptin-induced cell proliferation in ER^+^/PR^+^ cells (a, b). In triple-negative cells, leptin did not increase cell proliferation compared to the control (c, d). CQ treatment decreased cellular proliferation without leptin treatment in all cell lines tested at 48 h. This data indicates that autophagy is required for leptin-induced proliferation in ER^+^/PR^+^ and, also reflect the role of basal autophagy on the proliferation of breast cancer cells. The control was treated with a vehicle. C: control; L: leptin. n=2.


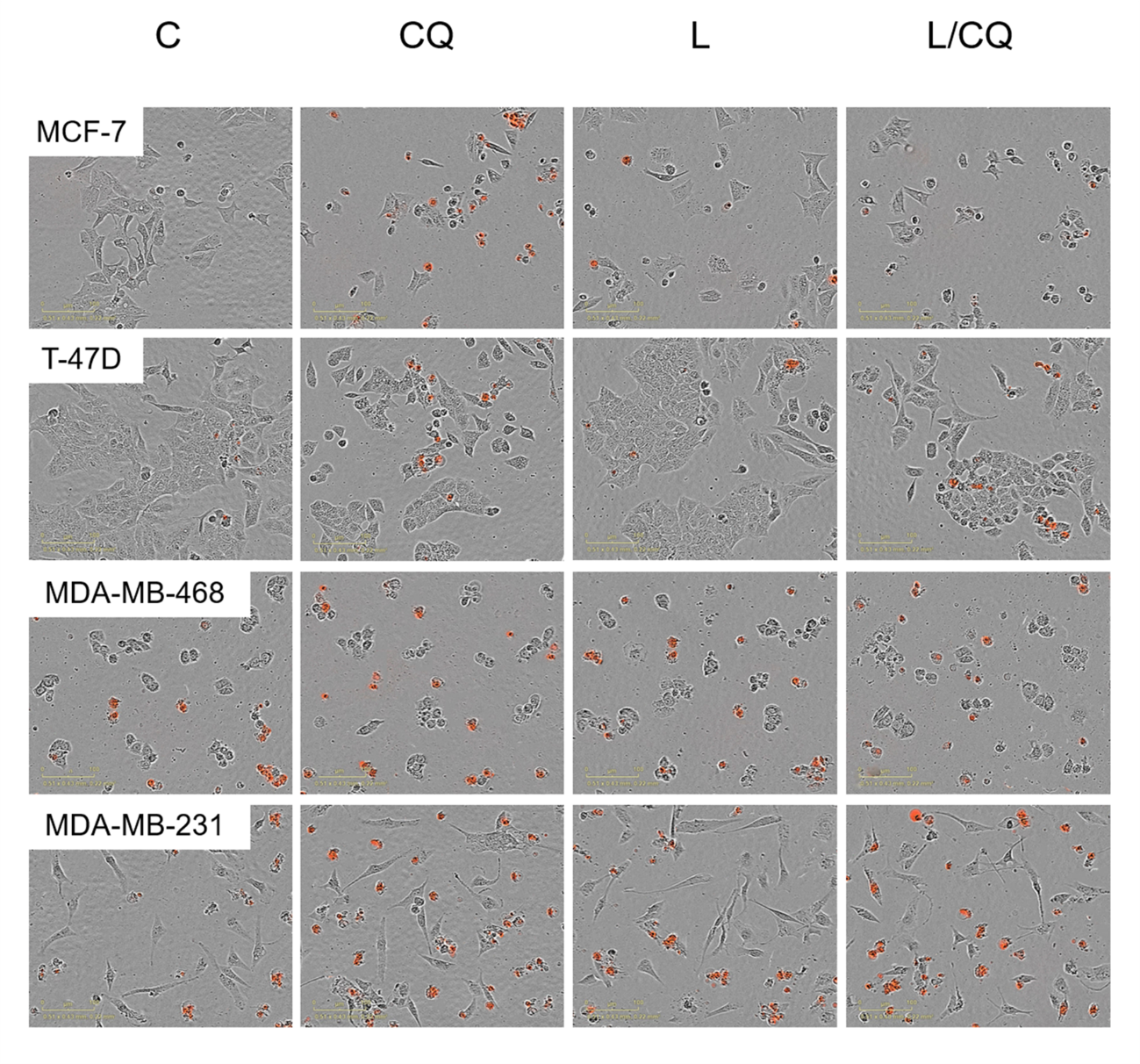


**Supplementary Figure S4. Effect of autophagy on cell death of breast cancer cells treated with leptin.** ER^+^/PR^+^ and triple-negative cells were treated with 400 ng/mL and 50 ng/mL of leptin respectively, for 48 h. After treatment, all cells were incubated with 10 μM propidium iodide (PI) for cell death assessment. Autophagy inhibition with chloroquine (CQ) did not increase the number of PI-positive cells relative to leptin (L) or control (C) treatment in MCF-7, T47-D or MDA-MB-468 cells. On the other hand, in MDA-MB-231 cells autophagy inhibition significantly increased cell death in control and leptin conditions. The control was treated with a vehicle. Images are representative of two independent experiments in quadruplicate.


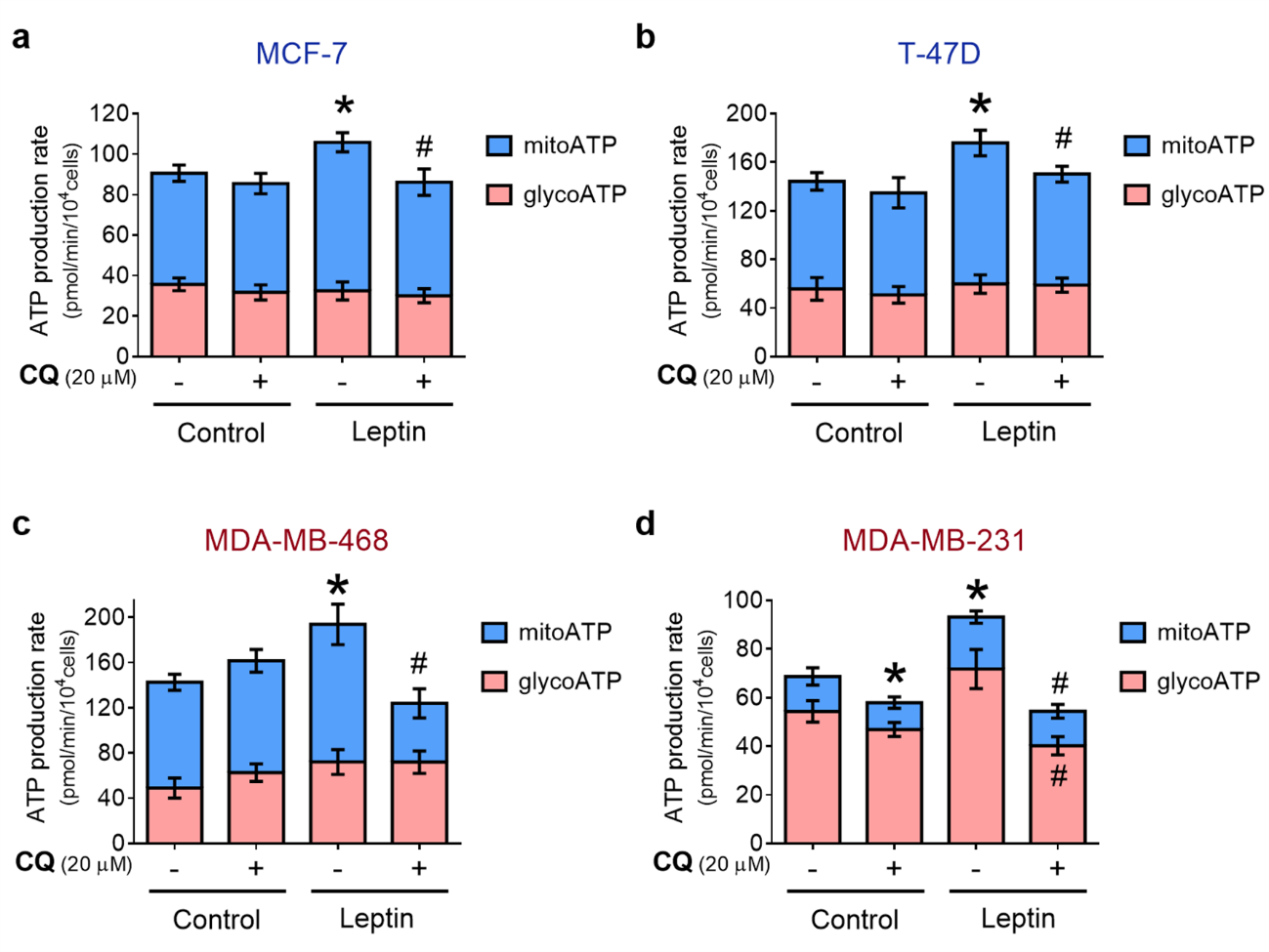


**Supplementary Figure S5. Effect of autophagy on energy metabolism of leptin-treated breast cancer cells at 48 h.** All cell lines were treated with leptin and/or chloroquine for 48 h. After the treatments, the metabolic change was measured. Data show that leptin increased mitochondrial ATP in all breast cancer cells (a-d). Interestingly, autophagy inhibition reduced the increase in mitochondrial ATP induced by leptin. On the other hand, in MDA-MB-231 cells, autophagy inhibition also prevented the increase in glycolytic ATP induced by leptin (d). The control in all experiments was treated with a vehicle. CQ: chloroquine. Graphs show mean ± S.D.; n=3 in triplicate; two-way ANOVA; Sidak post hoc; p<0.05. * vs C; # vs L.


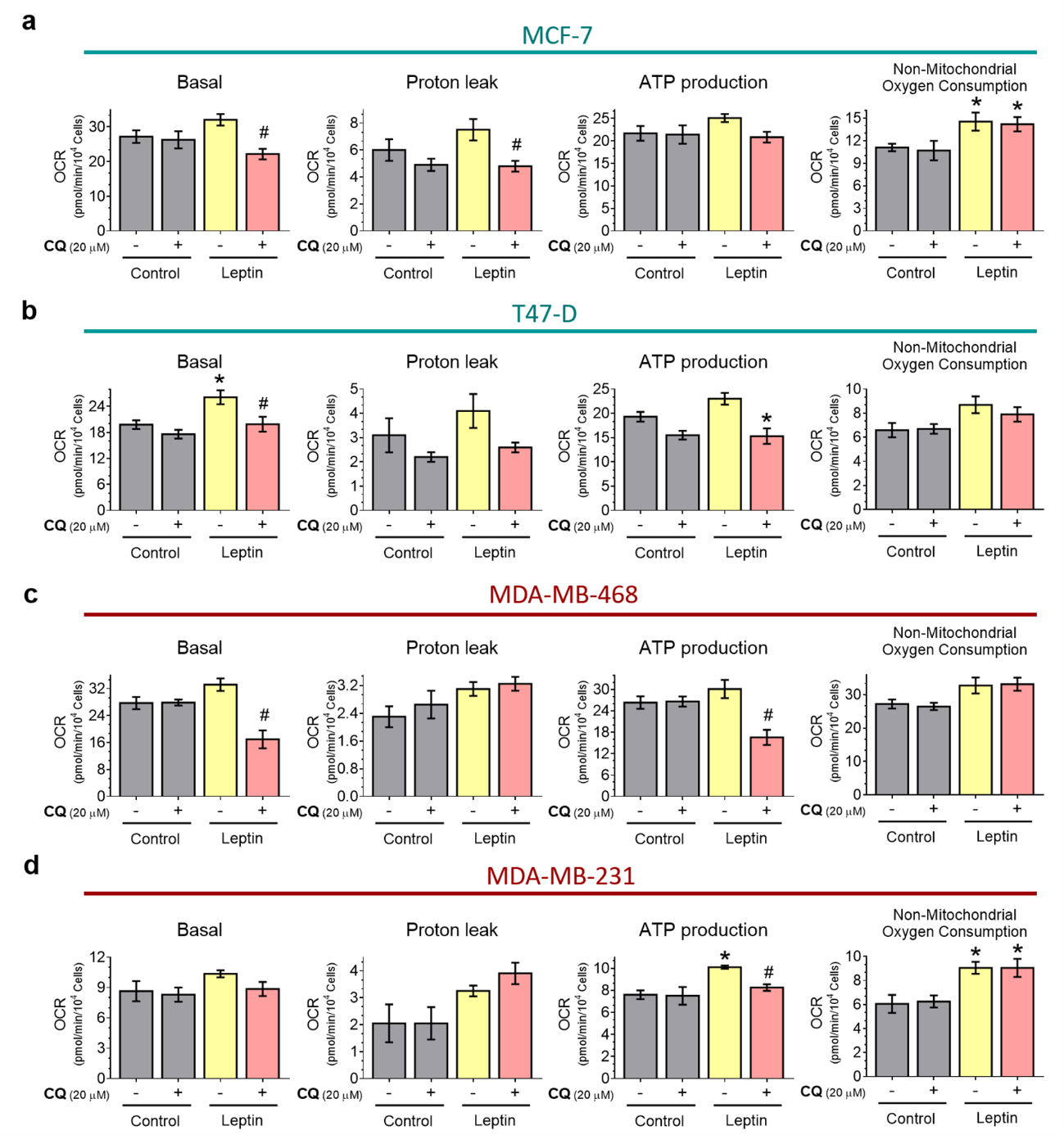


**Supplementary Figure S6. Effect of autophagy on mitochondrial function of leptin-treated breast cancer cells.** Cells were treated with leptin, CQ, or a mix of leptin and CQ for 24 h (a-d). Then, metabolic measurements were performed. In all cell lines treated with leptin, autophagy inhibition reduced mitochondrial basal OCR, proton leak and ATP production (a-d). Interestingly, non-mitochondrial oxygen consumption was increased by leptin treatment but was not regulated by autophagy. The control was treated with a vehicle. CQ: chloroquine. m ± s.d. n=3 in quadruplicate. One-way ANOVA. Tukey post hoc; p<0.05. * vs C; # vs L.


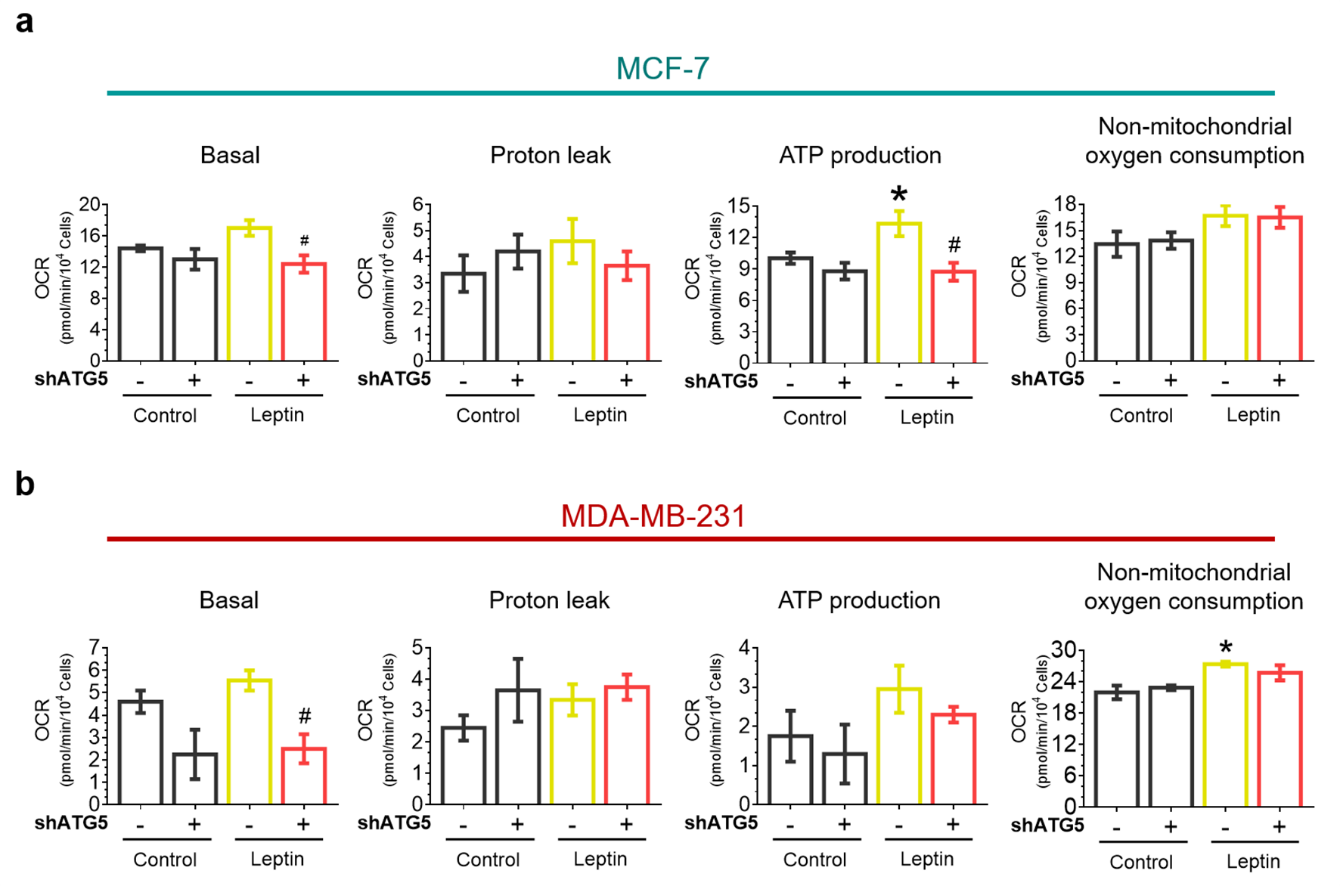


**Supplementary Figure S7. Effect of autophagy on mitochondrial function of leptin-treated breast cancer cells.** Lentivirally transduced cells were treated with leptin, for 24 h (a-b) and the metabolic measurements were performed. In MCF-7 cells, leptin increased mitochondrial ATP production and the knockdown of ATG5 reduced this parameter. In MDA-MB-231 cells, leptin increased non-mitochondrial oxygen consumption without changes by knockdown of ATG5 (b). Graphs show mean ± S.D. n=3 in quadruplicate. One-way ANOVA. Dunnett post hoc; p<0.05. * vs C; # vs L.


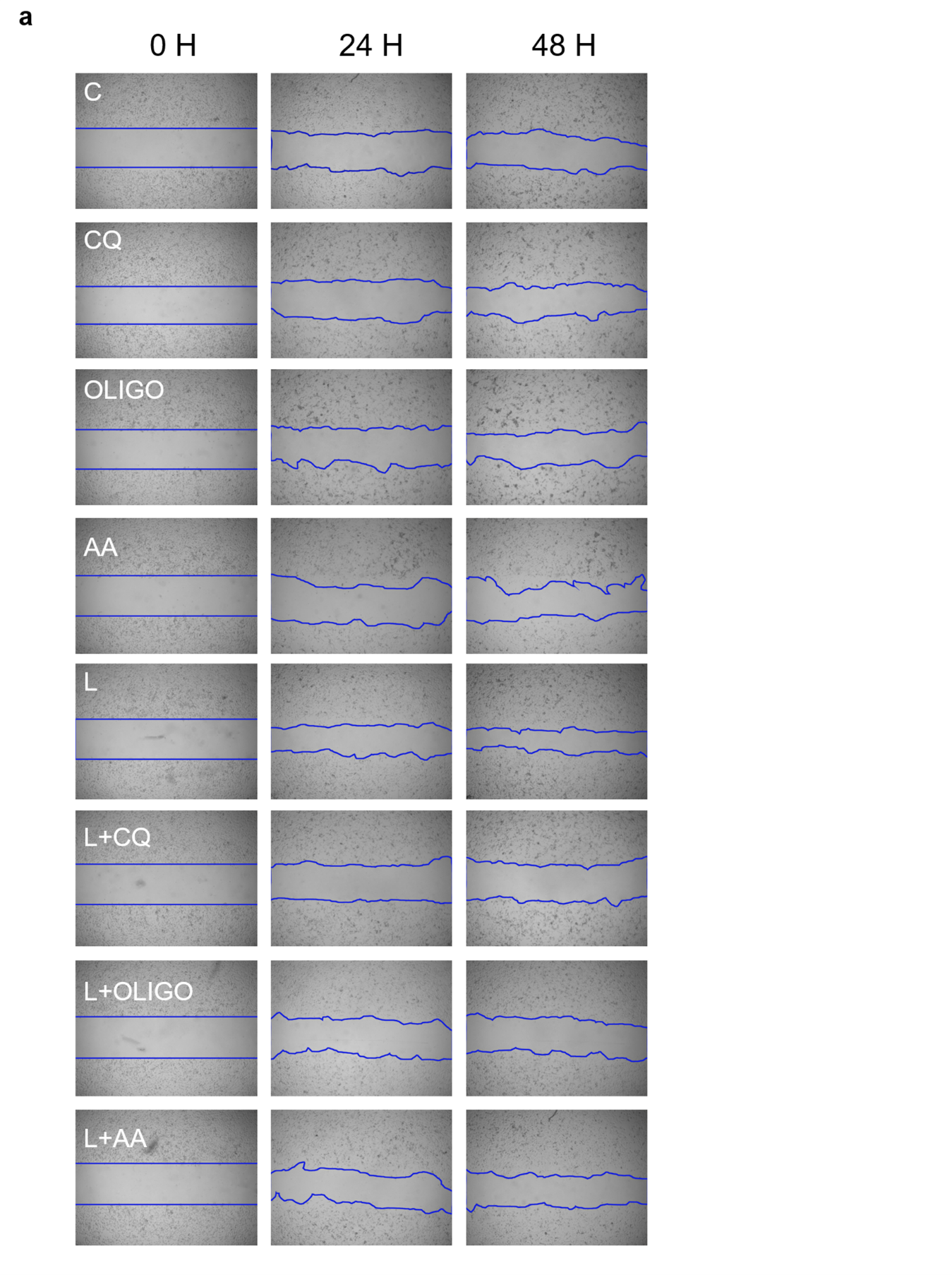


**Supplementary Figure S8. Effect of autophagy and mitochondrial inhibitors on cell migration of leptin-treated MDA-MB-231 cells.** Representative images of the wound healing assay. Leptin increased cell migration which was reduced by autophagy inhibition with CQ or mitochondrial inhibitors. C: control; L: leptin; CQ: chloroquine; OLIGO: oligomycin; AA: Antimycin A.


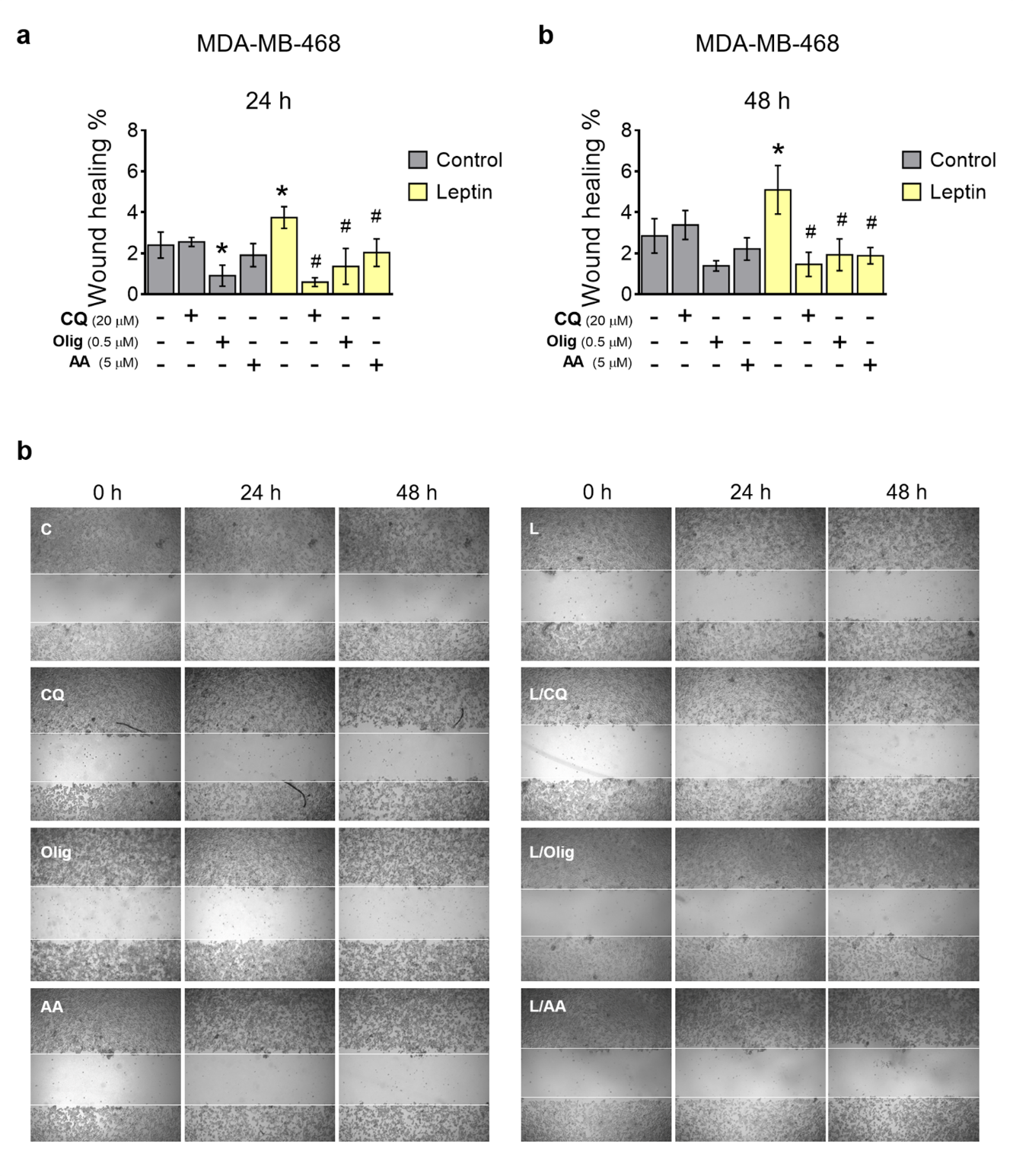


**Supplementary Figure S9. Effect of leptin, autophagy and mitochondrial metabolism on MDA-MB-468 cell migration.** Leptin induced cell migration at 24 (a) and 48 h (b). OXPHOS inhibition and autophagy inhibition reduced leptin-induced cell migration at 24 and 48 h (a, b). Importantly, basal cell migration was also decreased with CQ at 24 (a), but not at 48 h (b). Because leptin did not induce autophagy in MDA-MB-468 cells, the data suggests that basal autophagy is important to sustain the effect of leptin as an inducer of cell migration. Additionally, the data indicates that OXPHOS is necessary to sustain leptin-induced migration. Representative images of the wound healing assay are shown in panel c. Cells were treated with 50 ng/mL of leptin, 20 µM of CQ, Olig at 0.5 µM and AA at 5 µM for 48 h. The control was treated with a vehicle. C: control; L: leptin; CQ: chloroquine; Olig: oligomycin; AA: antimycin A. Graphs show mean ± S.D.; n=2 in triplicate; one-way ANOVA. Tukey post hoc; p<0.05. * vs C; # vs L.


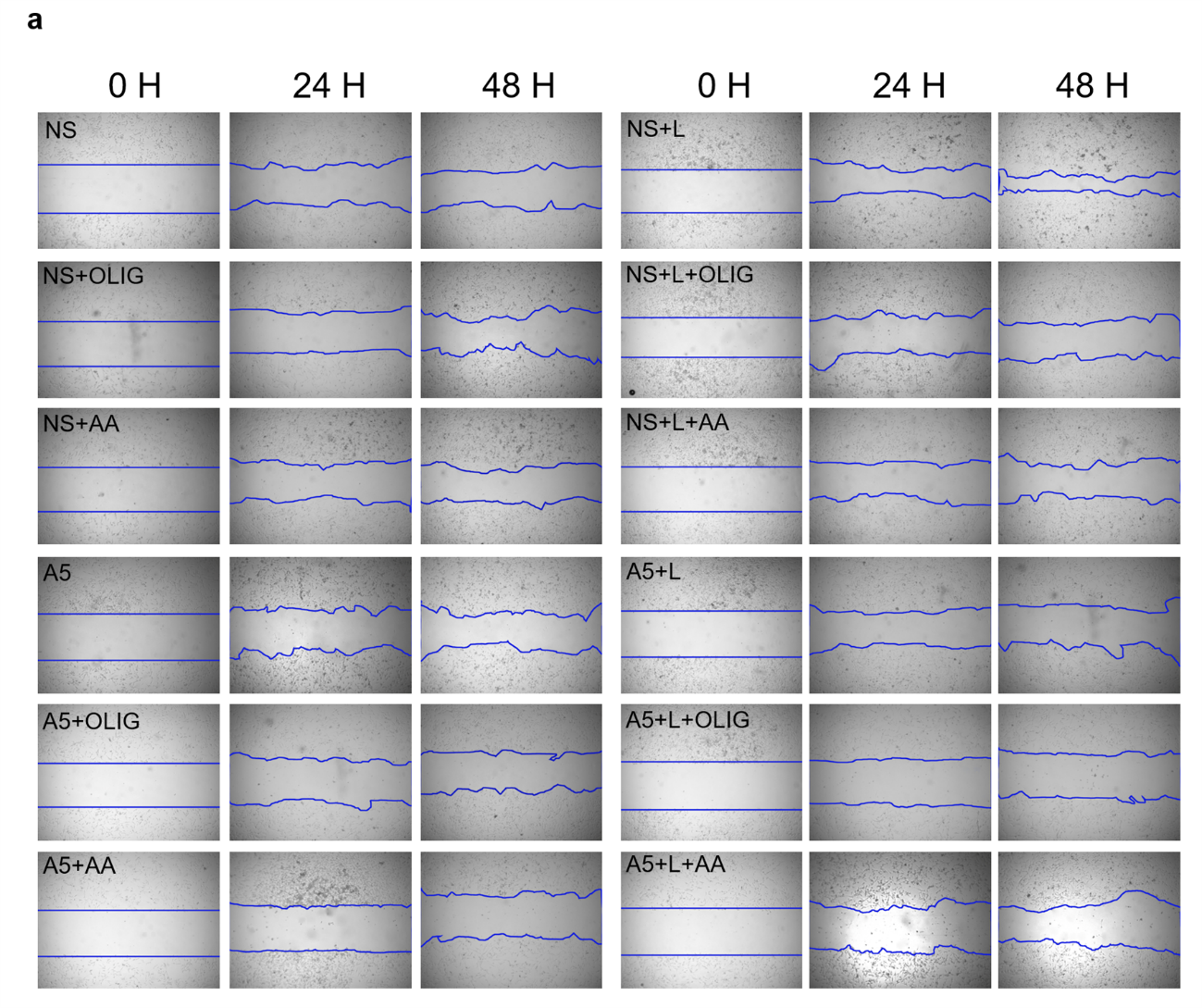


**Supplementary Figure S10. Effect of autophagy inhibition and mitochondrial function on leptin-induced migration in MDA-MB-231 cells.** Representative images of wound healing assay. Leptin increased cell migration in non-silencing cells and ATG5 knockdown reduced leptin-induced cell migration. Mitochondrial inhibitors also reduced leptin-induced cell migration although less efficiently than CQ. NS: non-silencing; A5: ATG5 knockdown; L: leptin; OLIG: oligomycin; AA: Antimycin A.

**
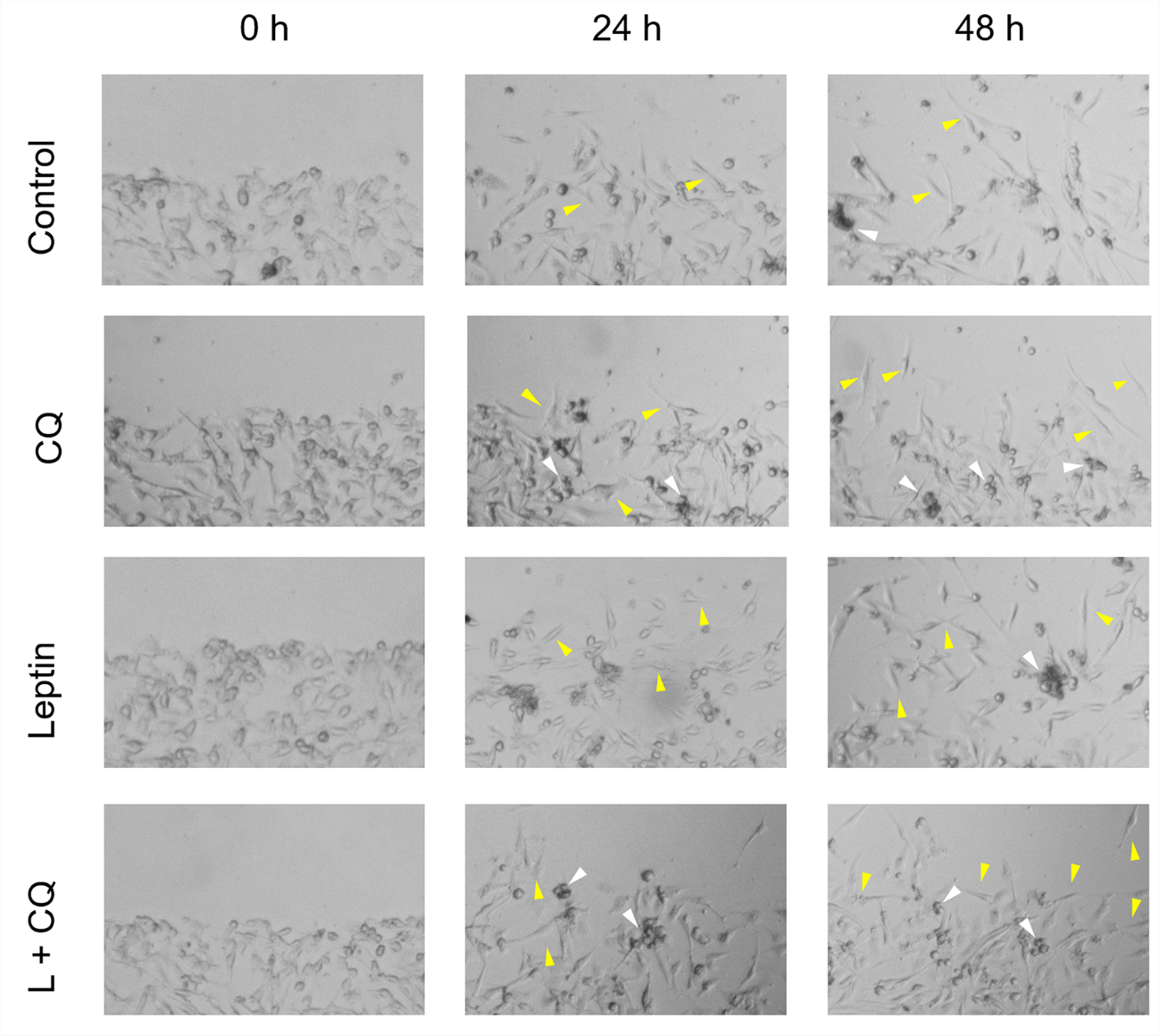
**

**Supplementary Figure S11. Representative images of the frontal wound healing assay in MDA-MB-231 cells.** The images show the migration front in the wound healing assay. The yellow arrows indicate migrating cells with fibroblastoid-like morphology and elongation of the needle-shaped membrane. The white arrows indicate clusters of cells with spheroid morphology, which correspond to dead cells. In all images, live cells in migration were observed. CQ: chloroquine; L: leptin. The images were cropped from the original image in Supplementary Figure S8.


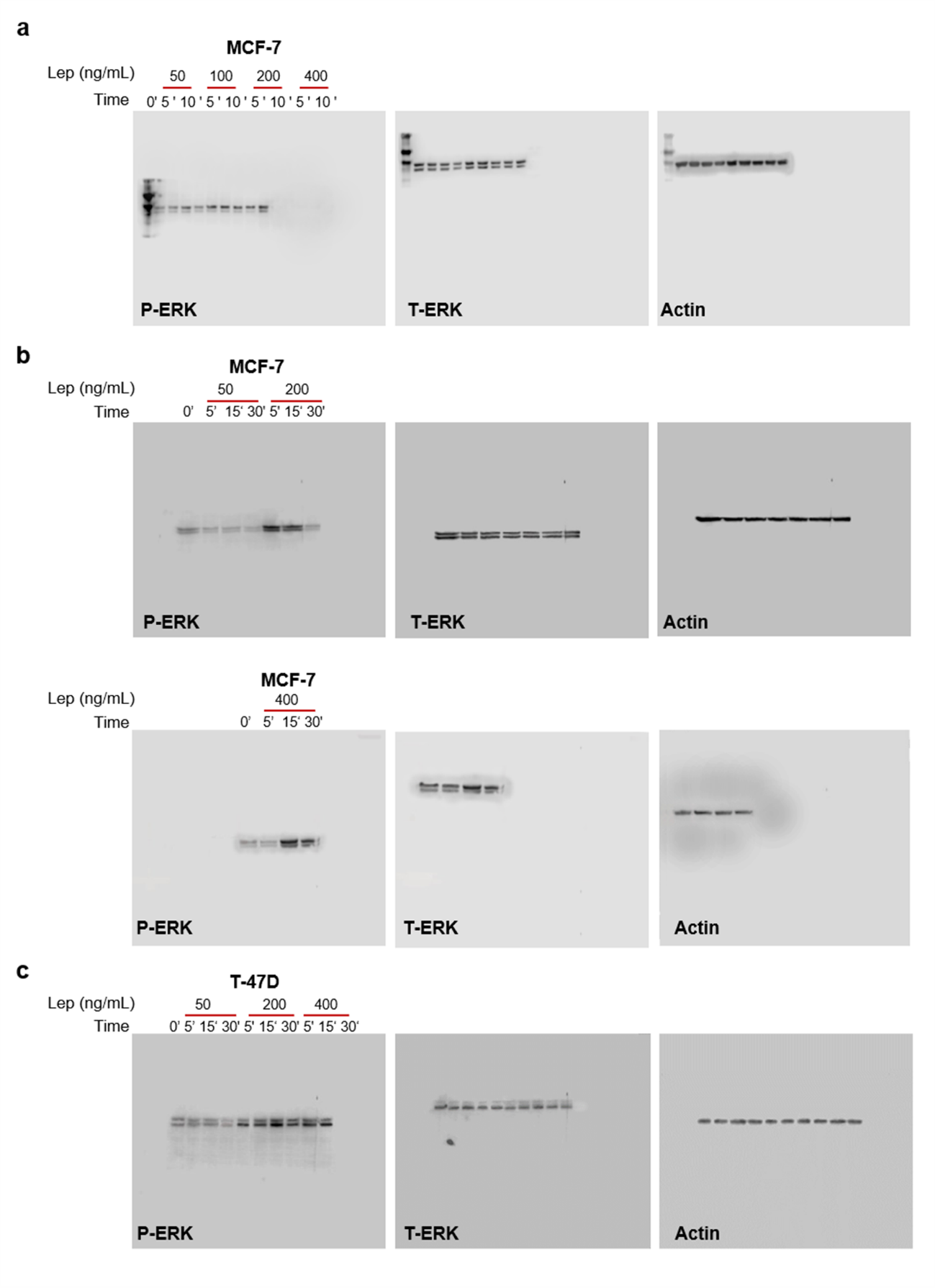


Continued…

**
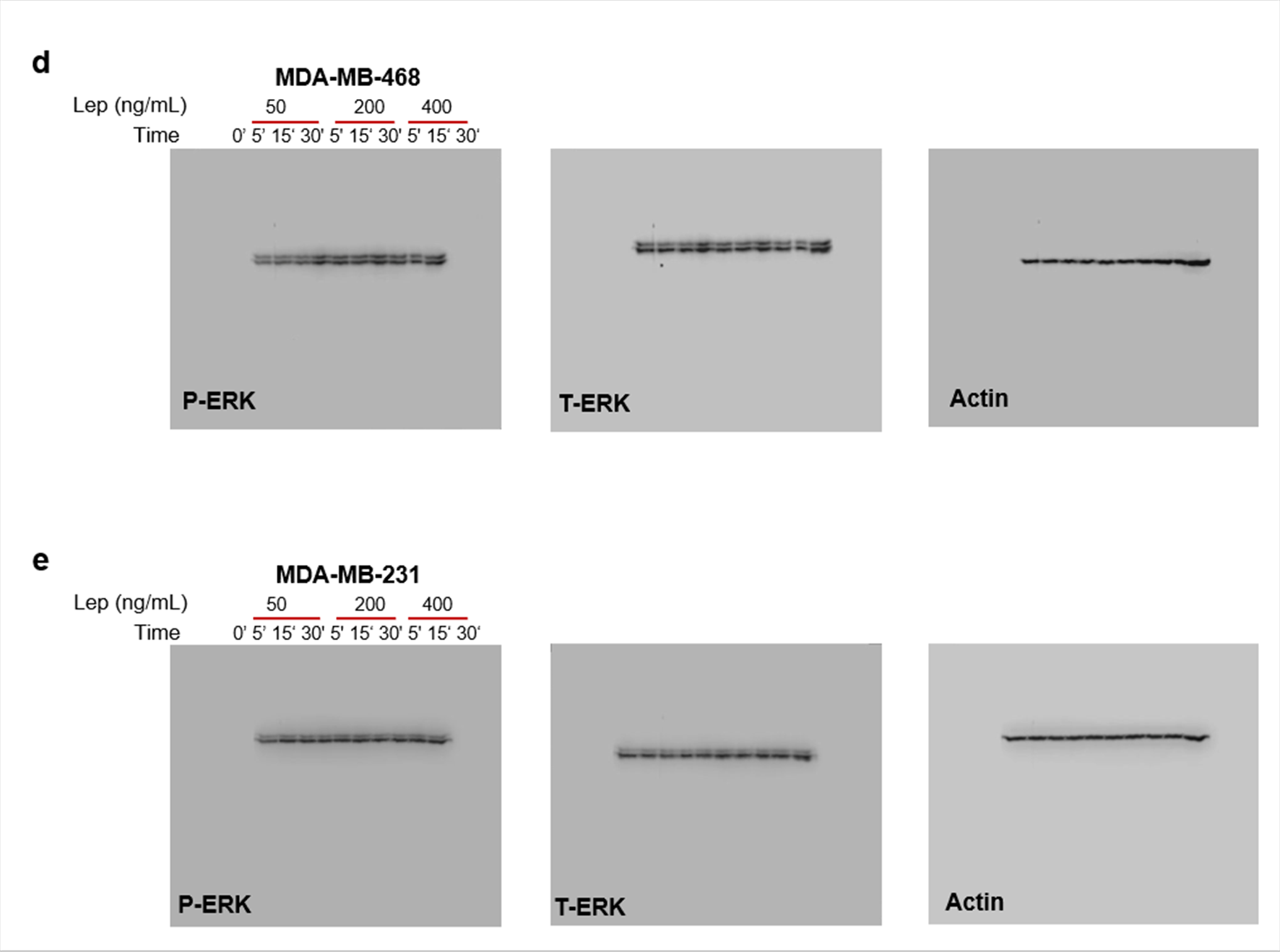
**

**Supplementary Figure S12. Original blots for phospho-ERK, total-ERK and β-actin in the main figure 1f-1i.** Panel **A** shows the first blot obtained from standardization for protein and antibody concentration, to revealed phosphor-ERK, total-ERK and β-actin. To identify all proteins in the molecular weight in blot revealing, we use MagicMark™ XP Western Protein Standard (LC5602). For MCF-7 cells leptin treatment with 400 ng/mL was revealed in separate membranes **(**b, down panel**)** with both membranes including a time cero control**.** In c, d and e panels, all leptin conditions were loaded and revealed in the same membrane by cell line. In all cases (a, b, c, d, and e), 30 µg of protein were resolved in 10 % SDS-PAGE.


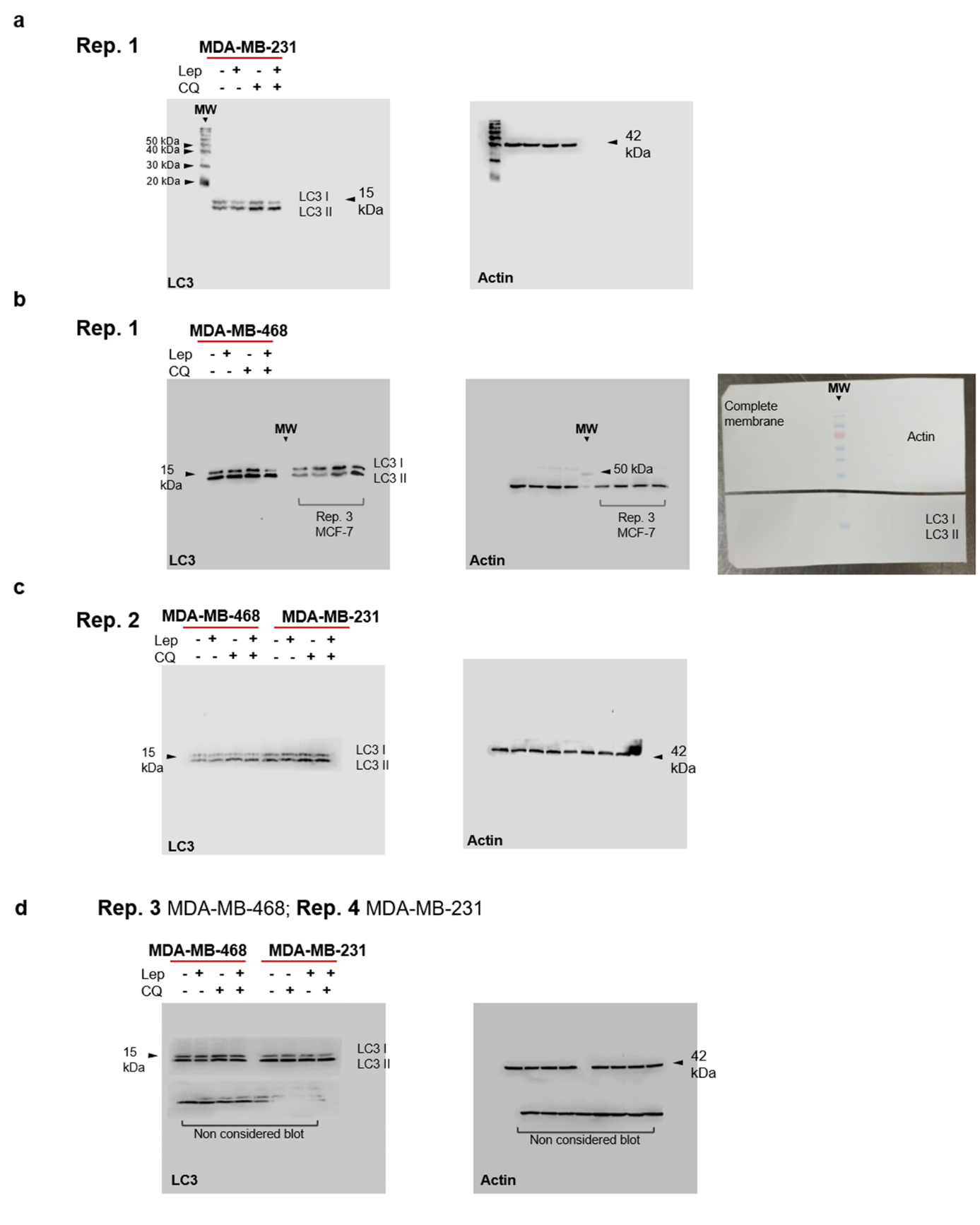


Continued…


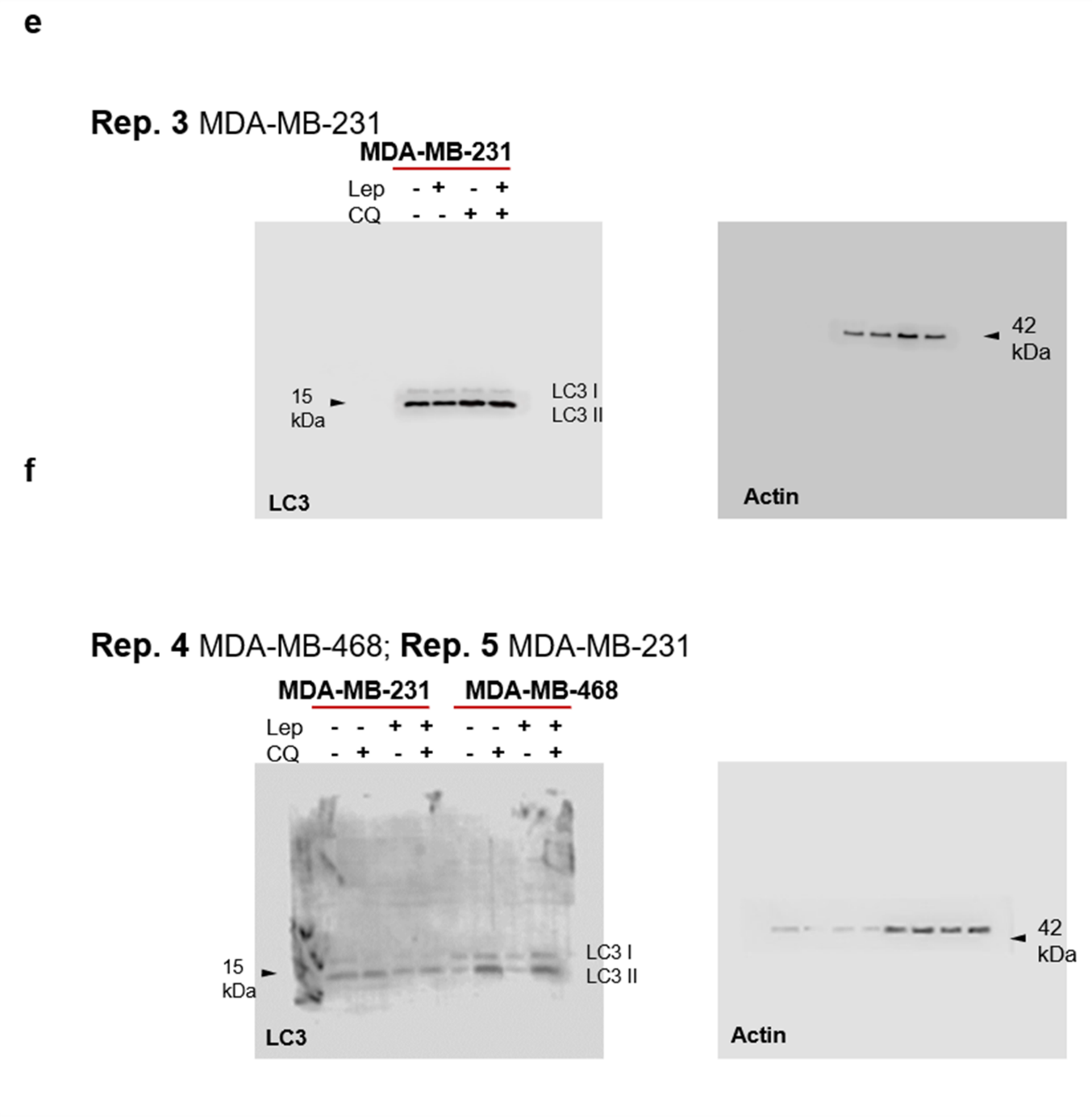


**Supplementary Figure S13. Original blots for LC3 and β-actin in all replicates of triple-negative breast cancer cells.** Panel a shows the first blot obtained for LC3 and β-actin in MDA-MB-231 cells. Both proteins were revelated in the same membrane, and we used MagicMark™ XP Western Protein Standard (Thermo-Fisher, LC5602) to identify the proteins in the corresponding molecular weight (a). Panel b corresponds to first replicate for MDA-MB-468 cells. Since the molecular weight of LC3 and actin differ, and we have previously validated these antibodies, we cut the membranes between 34 and 26 kDa (b, third image), after blocking with non-fat milk and previous to incubation with primary antibodies. In c, the second replicate for both triple-negative breast cancer cell lines are shown. In panels d and e the third replicate for triple-negative cells is shown. The samples of three replicas for each cell line were loaded in the next order: control, leptin, chloroquine, leptin/chloroquine (a-d). On the other hand, in the subsequent replicates the sample order was: control, chloroquine, leptin, leptin/chloroquine. The fourth replicate represented in panel d and f correspond to the main figure 2b. For all replicates 30 µg of protein were resolved in 15 % SDS-PAGE (a-e).


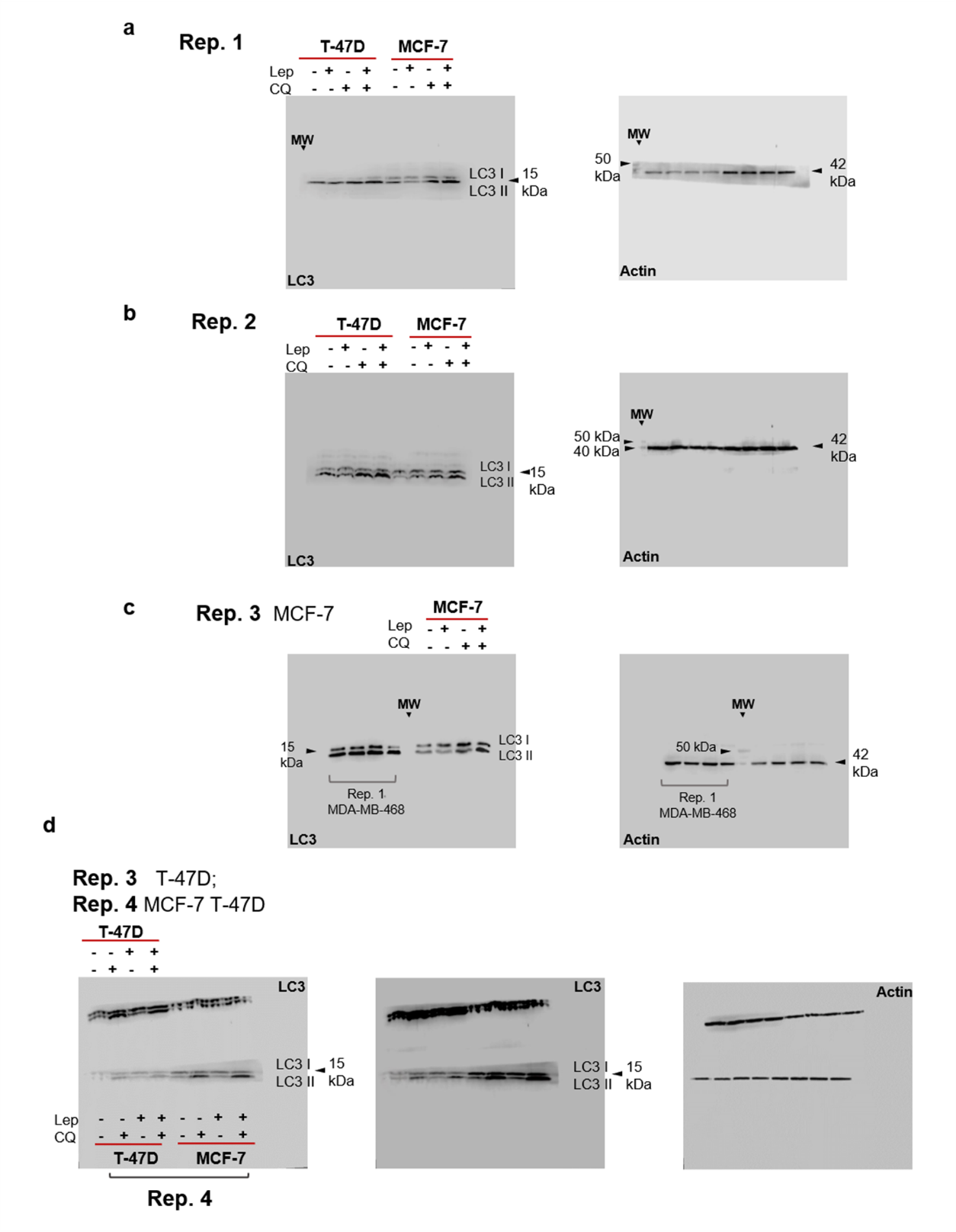


**Supplementary Figure S14. Original blots for LC3 and β-actin in all replicates of hormone-receptor positive breast cancer cells.** Membranes were cut after blocking with non-fat milk and before incubation with the primary antibodies (a-d). The first (a) and second (b) replicates for LC3 and β-actin in cell lines were loaded on the same gel. The third replicate for MCF-7 cells was solved together with a replicate of MDA-MB-468 cells (c). For replicates in a, b, and c the samples were loaded in the next order: control, leptin, chloroquine, leptin/chloroquine. Panel d shows the chemodetection of two membranes with different replicates, in which samples order were: control, chloroquine, leptin, leptin/chloroquine. The fourth replicate of hormone-receptor positive cells corresponds to main figure 2a. For all conditions, 30 µg of protein were resolved in 15 % SDS-PAGE (a-d).


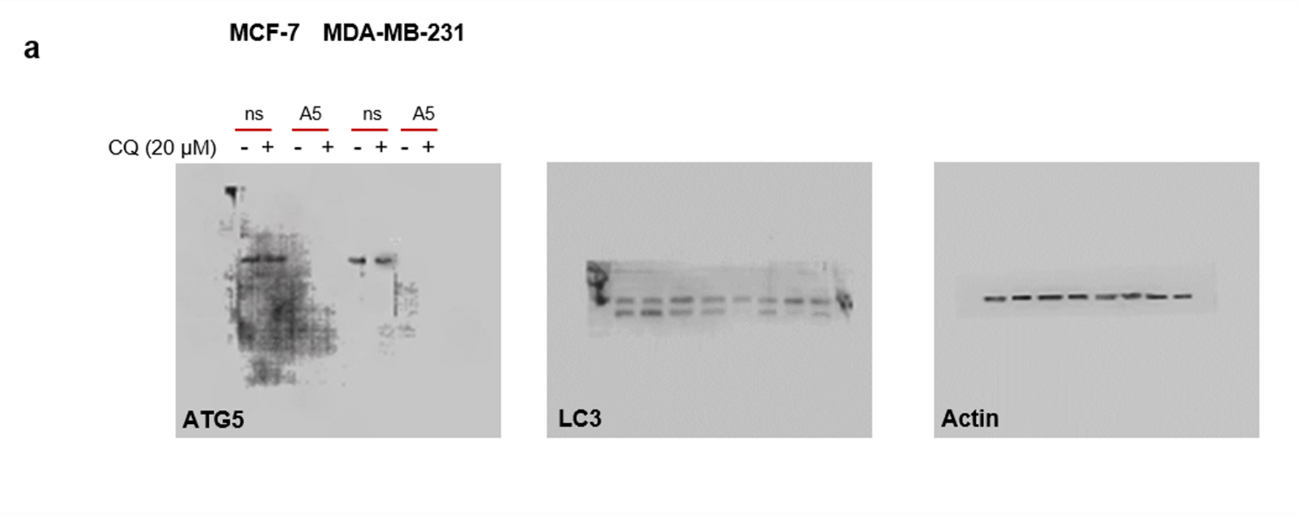


**Supplementary Figure S15. Original blots for ATG5, LC3 and β-actin correspond to main figure 4e.** First, Detection of ATG5 was performed on the complete (a). The chemodetection showed a single intense band corresponding to ATG5. For detection of LC3 (b) and β-actin (c), the membrane was cropped as previously specified. 30 µg of protein were resolved in 12 % SDS-PAGE.


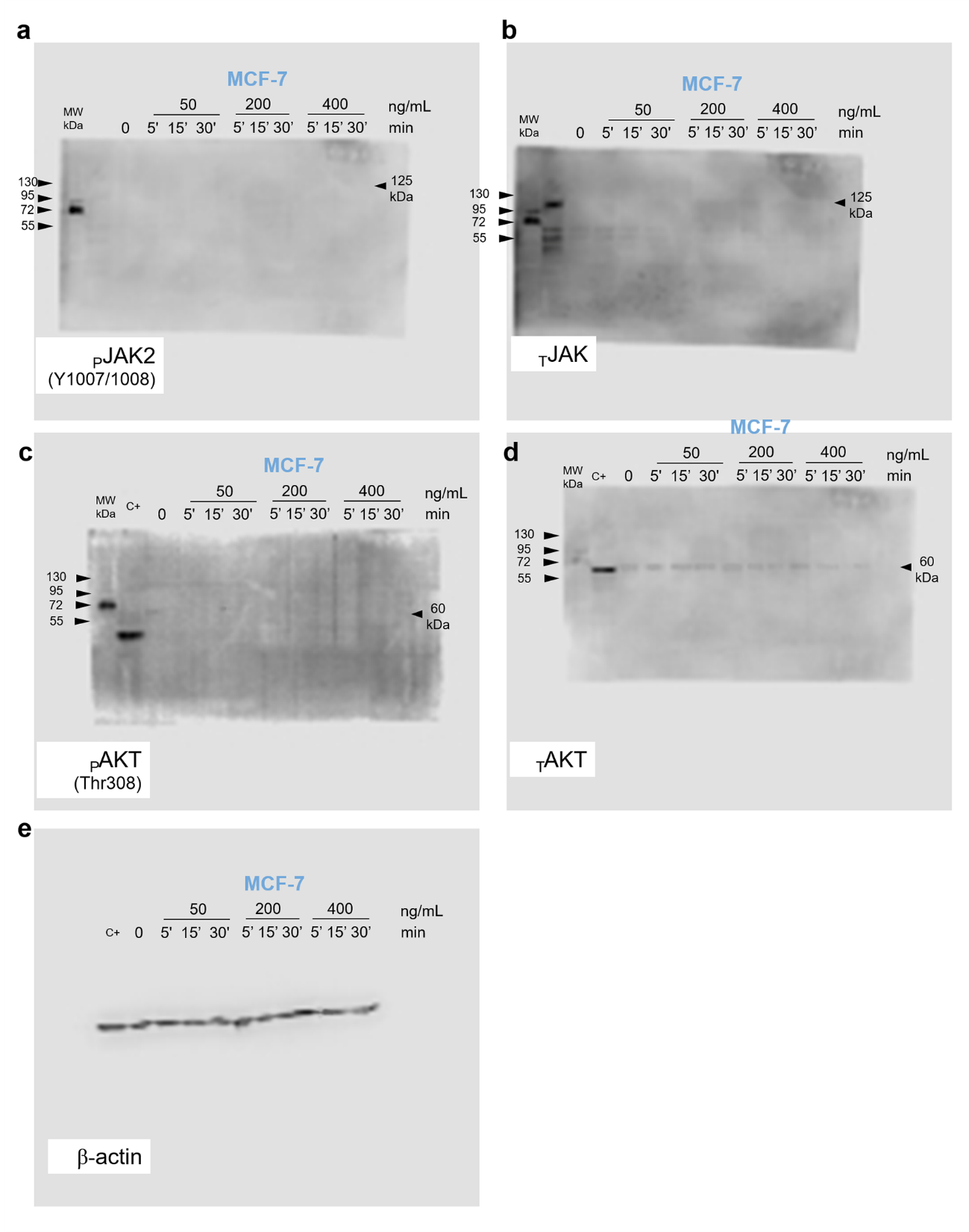


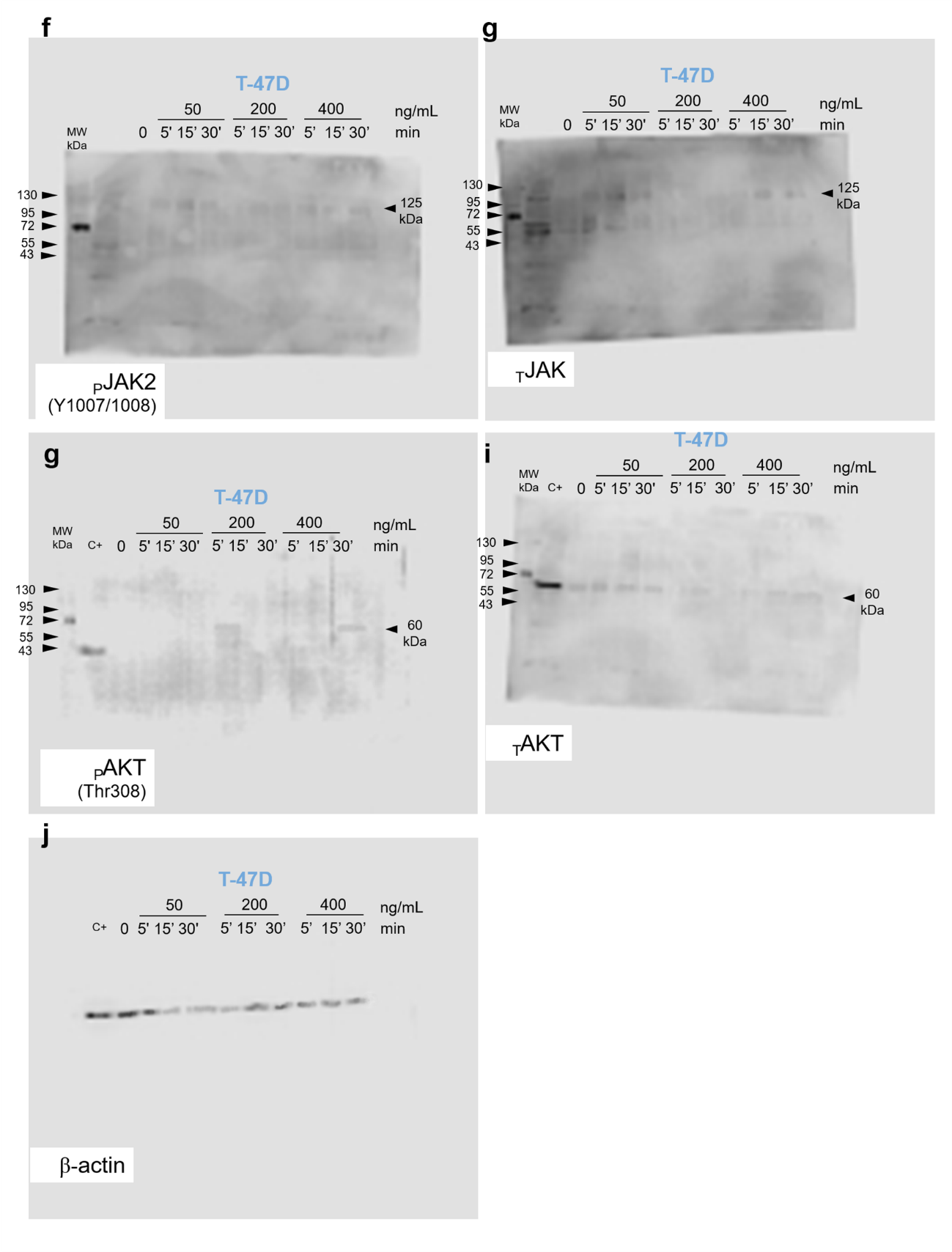


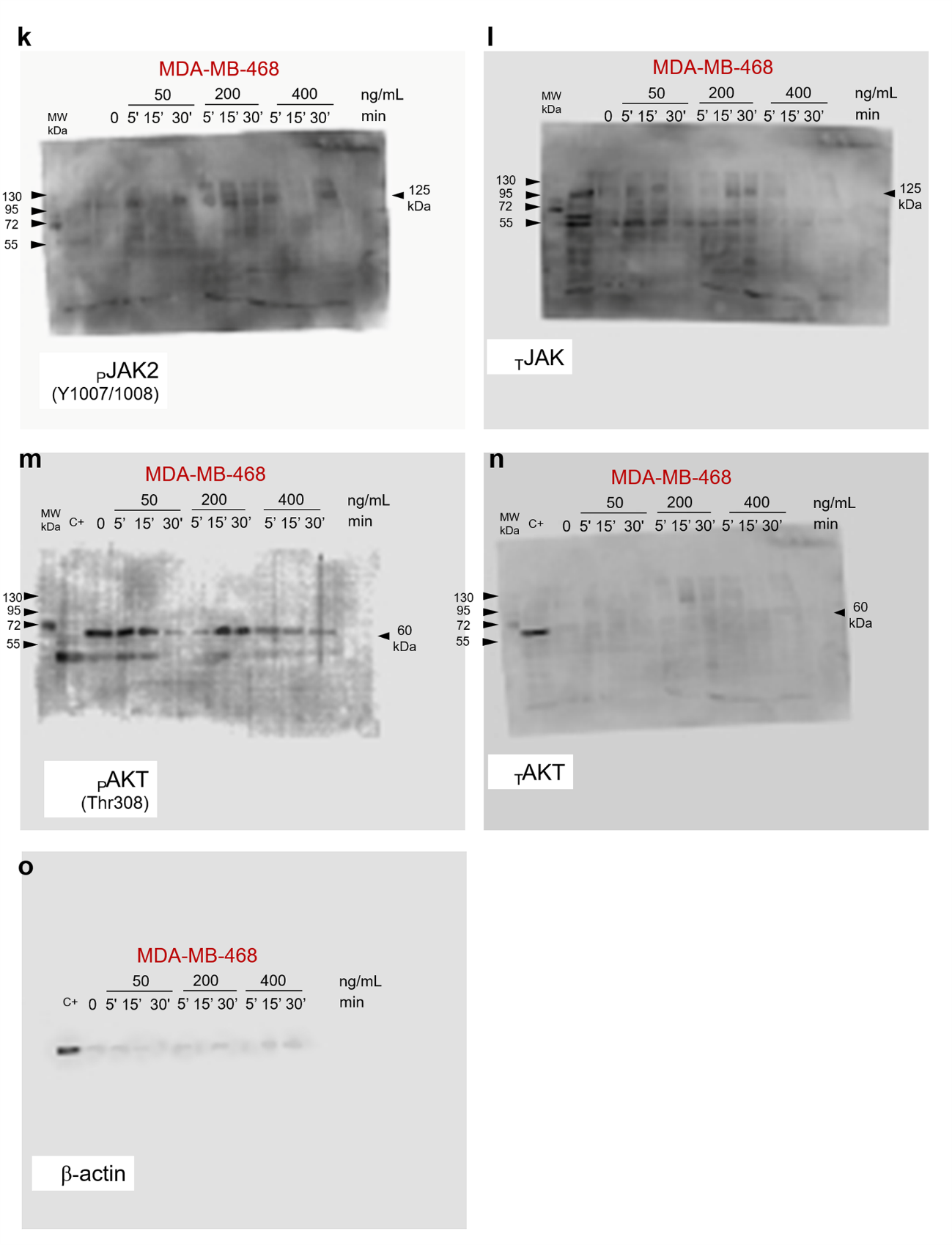


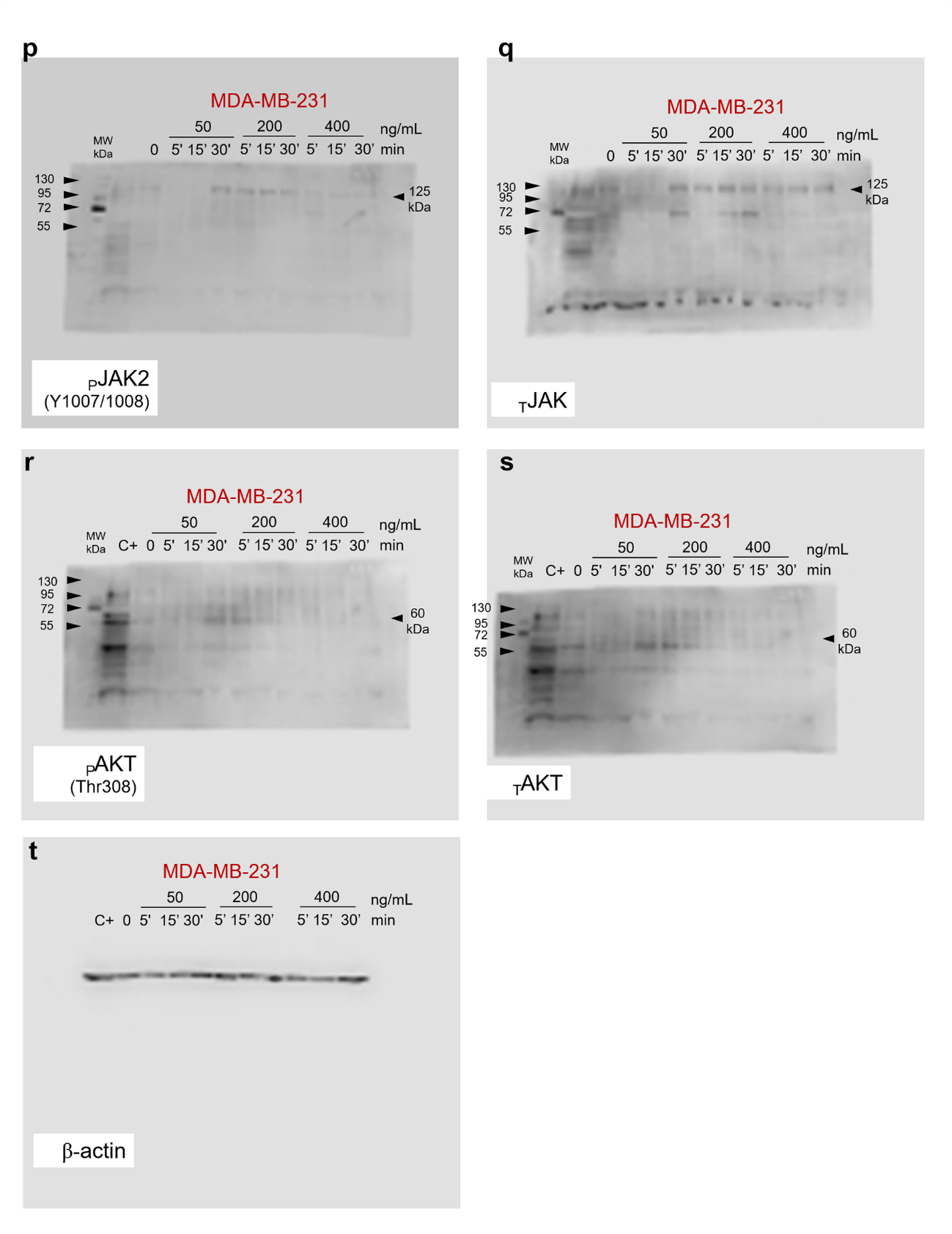


**Supplementary Figure S16. Original and complete blots for phospho-JAK, total-JAK, phospho-AKT, total-AKT and β-actin in breast cancer cells treated with leptin.** Cells were treated with 50, 200 and 400 ng/mL of leptin during 5, 15 and 30 minutes. All chemodetection of proteins was done on the same membrane per cell line. For all conditions, 30 µg of protein were resolved in 12 % SDS-PAGE. Protein extract from MCF-7 cells maintained in 10 % FBS and 10 µg/mL insulin supplemented-media was used as a positive control for phospho-AKT and total-JAK (C^+^).
